# Optogenetic regulation of site-specific subtelomeric DNA-methylation

**DOI:** 10.18632/oncotarget.10394

**Published:** 2016-07-04

**Authors:** Samrat Roy Choudhury, Yi Cui, Anoop Narayanan, David P. Gilley, Nazmul Huda, Chiao-Ling Lo, Feng C. Zhou, Dinesh Yernool, Joseph Irudayaraj

**Affiliations:** ^1^ Department of Agricultural & Biological Engineering, Bindley Bioscience Center, Purdue Center for Cancer Research, Purdue University, West Lafayette, IN 47907, USA; ^2^ Bindley Laboratory of Structural Biology, Bindley Bioscience Center, Purdue University, West Lafayette, IN 47907, USA; ^3^ Department of Medical and Molecular Genetics, Indiana University School of Medicine, Indianapolis, IN 46202, USA; ^4^ Department of Anatomy and Cell Biology, Indiana University School of Medicine, Indianapolis, IN 46202, USA; ^5^ Stark Institute of Neuroscience Research, Indiana University School of Medicine, Indianapolis, IN 46202, USA

**Keywords:** optogenetics, single-cells tools, subtelomeric DNA-methylation, telomere-elongation

## Abstract

Telomere length homeostasis, critical for chromosomal integrity and genome stability, is controlled by intricate molecular regulatory machinery that includes epigenetic modifications. Here, we examine site-specific and spatiotemporal alteration of the subtelomeric methylation of CpG islands using optogenetic tools to understand the epigenetic regulatory mechanisms of telomere length maintenance. Human DNA methyltransferase3A (DNMT3A) were assembled selectively at chromosome ends by fusion to cryptochrome 2 protein (CRY2) and its interacting complement, the basic helix loop helix protein-1 (CIB1). CIB1 was fused to the telomere-associated protein telomere repeat binding factor-1 (TRF1), which localized the protein complex DNMT3A-CRY2 at telomeric regions upon excitation by blue-light monitored by single-molecule fluorescence analyses. Increased methylation was achieved selectively at subtelomeric CpG sites on the six examined chromosome ends specifically after blue-light activation, which resulted in progressive increase in telomere length over three generations of HeLa cell replications. The modular design of the fusion constructs presented here allows for the selective substitution of other chromatin modifying enzymes and for loci-specific targeting to regulate the epigenetic pathways at telomeres and other selected genomic regions of interest.

## INTRODUCTION

Telomeres disguise chromosome ends preventing aberrant double strand break repair [1]. The defects in telomere maintenance cause end-to-end chromosome (telomere) fusions, which are well known sources of genomic instability and subsequent cancer development [2-4]. Additionally, telomere fusions and stochastic breakage-fusion cycles caused by dysfunctional telomeres are considered a likely source of genomic heterogeneity seen in many human cancers [5, 6]. More specifically, telomeres are specialized nucleoprotein complexes composed of multiple arrays of duplex TTAGGG repeats and a variety of telomere-associated proteins [1, 7]. Telomere repeat binding factor-1 (TRF1) and 2 (TRF2) along with TIN2, POT1, TPP1 and RAP1 form the shelterin complex, which cap or protect the chromosome ends from deleterious telomere fusions [7-10]. TRF1 binds directly to telomeric and subtelomeric interstitial TTAGGG repeat sequences as a homodimer and helps to maintain telomere length and stability by exerting a cis-acting inhibition on telomerase elongation [11, 12]. Telomerase is the ribonucleoprotein enzyme responsible for polymerization of telomeric DNA required to overcome the end-replication problems associated with conventional cellular DNA polymerase [13]. Several reports indicated that progressive shortening of telomeric DNA in telomerase positive tumors were correlated with increasing levels of TRF1 [14]. Increased levels of TRF1 did not alter the catalytic activity of telomerase but was proposed to negatively regulate telomere length through steric exclusion of telomerase access to chromosome ends [15].

Telomeres and adjacent subtelomeric sequences are generally considered to be regions of heterochromatin architecture [16, 17]. Similar to other heterochromatic regions, subtelomeric chromatin contains several marks of histone modifications and dense DNA methylation [18-20]. Subtelomeres are enriched with gene promoters, segmental duplications, satellite sequences or telomere-like interstitial repeat sequences [21, 22]. Ectopic epigenetic changes at the subtelomeric regions have been proposed to influence several biological pathways or induce cellular reprogramming, a detailed mechanism of which is yet to be understood [23]. Epigenetic modifications at subtelomeres have also been recognized to play an important role in telomere integrity and length regulation [21, 24]. For example, telomerase expressing cancer cells retain a high level of DNA methylation at the subtelomeres to maintain their heterochromatic state through repression of telomeric-repeat containing transcript (TERRA) [25]. In addition, inhibition of global DNA demethylation, which results from triple-negative knockout (TKO) of ten-eleven translocation methylcytosine dioxygenase (TET) proteins, resulted in the elongation of telomere length in mouse embryonic stem cells [26]. Furthermore, another line of studies reported the positive/negative or no correlation between subtelomeric methylation and telomere length in different disease models [27, 28]. In summary, studies show that DNA methylation of subtelomeric regions and epigenetic enzymes play a crucial role in regulation of telomere length [29]. However, to date, the effect of targeted increase in subtelomeric DNA methylation and its effect on telomere length maintenance have not been achieved due to the inability to localize methyltransferase activity particularly in the site-specific subtelomeric regions.

Recent discoveries of optically inducible (optogenetic) proteins of plant or microbial origin offer tunable methods to modulate the dynamic/kinetic nature of specific molecular interactions with spatiotemporal precision [30-32]. Herein, we demonstrate active and on demand targeting of DNMT3A at the subtelomeric DNA regions to increase methylation marks at these specific genomic locations in HeLa cells. The optically inducible dimerizing protein pair CRY2-CIB1 was fused to DNMT3A and TRF1 respectively for this targeting strategy. Single molecule fluorescence tools are introduced to demonstrate and monitor these optogenetically-induced interactions in live single cells. Fluorescence Lifetime Imaging Microscopy-based Förster Resonance Energy Transfer (FLIM-FRET) analysis was used to determine their dynamic intra-nuclear distribution patterns. We find that the optogenetic regulatory platform developed here can specifically alter the endogenous subtelomeric epigenetic status selectively, enabling a process that is facile, tunable, and precise. This platform provides a biophysical and biochemical route to understand epigenetic modulation of subtelomeric methylation and its role in telomere length maintenance. In this study, we report the effect of light-induced association of optogenetic fusion proteins on selective increase in DNA methylation levels at subtelomeric regions at the chromosomal ends that results in telomere lengthening.

## RESULTS

### Construction and expression of optogenetic fusion proteins, designed to target the subtelomeric regions

**Figure 1:**
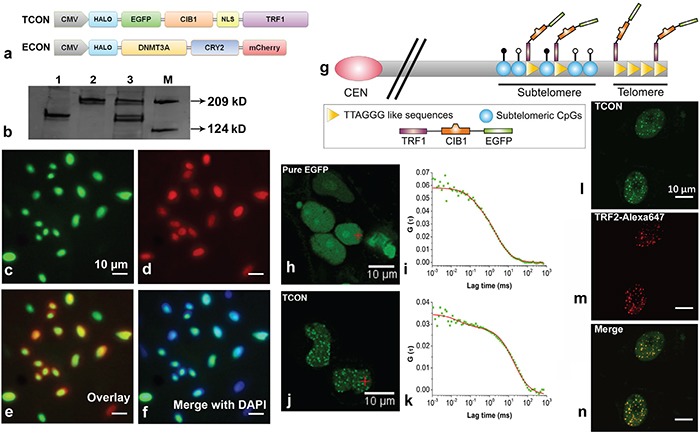
Telomere targeting of TCON and nuclear co-localization with ECON **a.** Schematic of the HALO tagged TCON and ECON fusion proteins, **b.** Western blot analysis demonstrates production of intact TCON (~149 kD; lane-1), ECON (~223 kD; lane-2), and TCON plus ECON (lane-3) fusion proteins in HeLa cells. Representative images showing the nuclear localization of TCON **c.** ECON **d.** their co-localization **e.** and their overlap with DAPI stained nucleic acids **f.** **g.** Schematic showing the binding of TCON to the telomeric repeat sequences through TRF1. FCS measurements showed the distinct distribution and diffusion pattern of TCON tagged EGFP **j, k.** in comparison to the control EGFP **h, i.** Co-localization of TCON and TRF2 is revealed using the overlapping fluorescence of EGFP of TCON and Alexa-647 TRF2 (m) antibody bound to TRF2 **l, m** and **n.** in HeLa cells. Because TRF2 is telomere specific binding protein, the co-localization of TCON to the same locus reveals the association of TCON with telomeres.

We designed two constructs expressing fusion proteins namely the telomere target construct (TCON) and the epigenetic effector construct (ECON) containing fluorescent reporters EGFP and mCherry respectively (Figure 1a). Additionally, TCON contains the human TRF1 linked to the wild type CIB1 (cryptochrome-2-interacting-basic-helix-loop helix) protein, whereas DNMT3A and CRY2PHR (N-terminal photolyase homology region domain of CRY2) proteins are present in ECON. The optically active CIB1 has a blue-light dependent reversible association with CRY2PHR [33]. The transient expression of TCON and ECON in HeLa cells resulted in the production of full-length fusion proteins of 149 and 223 kDa mass respectively (Figure 1b). Transient co-transfection efficiency with TCON and ECON reached up to 70 % in HeLa cells as revealed by fluorescence-activated cell sorting (Supplementary Figure 1g) analysis. Per design, when expressed individually, TCON localized predominantly in the nucleus (Figure 1c), while the ECON was expressed in both the cytoplasm and nucleus (Supplementary Figure 1b, 1d). Co-expression of the fusion proteins from nucleus were also evidenced by fluorescence imaging and DAPI-nucleic acid staining (Figure 1e, 1f). Next, we asked whether the expressed TCON would bind to the telomeres, using Fluorescence Correlation Spectroscopy (FCS), co-localization analysis with TRF2 antibody, and a chromatin immunoprecipitation (ChIP) assay. A distinct, punctate pattern of EGFP florescence was observed in TCON transfected cells compared to the diffused distribution in the control cells transfected with pure EGFP (Figure 1h and 1j). Two species, one diffusing slowly (bound component) and the other rapidly (free component) with diffusion coefficients of 5.96 ± 0.58 and 0.92 ± 0.08 μm^2^/s respectively were observed in TCON positive cells (Figure 1k). The fraction of bound TCON in the punctate loci was estimated to be 83.0 ± 17.0 %. By contrast, a single freely diffusing species with a diffusion coefficient of 11.23 ± 2.06 μm^2^/s was observed in control cells expressing EGFP (Figure 1h, 1i). FCS data suggests that majority of the TCON was in a bound state, possibly to the telomeres through TRF1. To confirm the binding of TCON to the telomeres, a co-immunostaining assay was performed by utilizing the fluorescence from EGFP-TCON construct and a fluorescent antibody that binds to the endogenous TRF2 proteins (a bona fide telomere binding protein [15]. We observed a typical, punctate, and overlapping staining pattern, which confirmed the co-localization of TRF1 (derived from TCON constructs) and TRF2 at the telomeric foci (Figure 1l, 1m, 1n). To further demonstrate specific association of TCON to telomeres, a ChIP assay was conducted by isolating DNA bound to TCON by immunoprecipitation using anti-GFP antibody. Using the isolated DNA as template and telomere specific primers, a 123 bp amplicon was obtained by PCR, whereas the negative control showed no detectable amplicons (Supplementary Figure 2). Overall our results show that full-length TCON localizes to the nucleus, and a majority of these binds to the telomeres and subtelomeric loci characterized by TTAGGG sequences.

### Optically induced association of TCON and ECON

**Figure 2:**
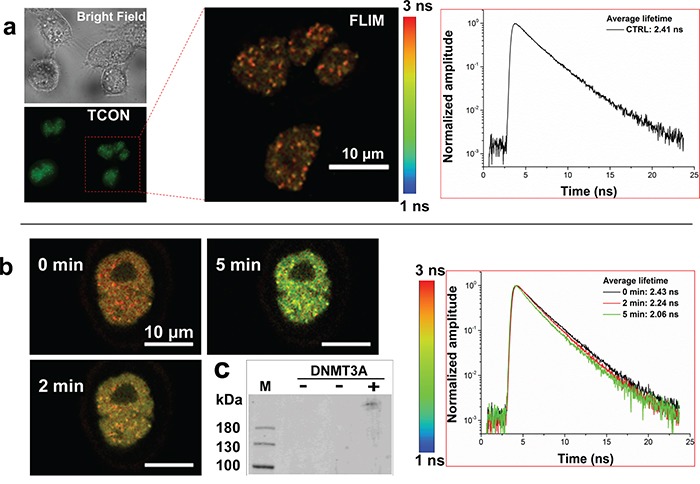
Blue light induced association of optogenetic fusion proteins **a.** Fluorescence lifetime analysis of EGFP component of TCON fusion protein in transiently transfected HeLA cells using FLIM-FRET. Fluorescence lifetime was estimated to be 2.41 ns. **b.** Blue-light induced change in fluorescence lifetime of EGFP component of TCON fusion in the presence of ECON. Representative HeLA cells co-transfected with TCON and ECON constructs was exposed to 8 mW/cm^2^ of blue light for the indicated time periods. Fluorescence lifetime decreases from 2.43 ns to 2.06 ns over a 5 min exposure to blue light, due to the FRET between EGFP and mCherry, validating the optically induced association of CRY2PHR and CIB1 that are components of TCON and ECON fusions respectively. **c.** Western blot analysis of co-immunoprecipitated fraction from cells transfected with either TCON, or TCON and ECON treated with presence (+) and in the absence (-) of blue light. Immunoprecipitation was carried out using EGFP antibody, whereas DNMT3A antibody was used for the detection of pulled-down ECON in Western blot. An ECON specific band (*ca.* 223 kDa) was observed in the blot in the protein fractions of co-transfected illuminated cells (lane-3). In contrast, no anti-DNMT3A Ab specific band was detected in cells transfected with TCON (lane-1) or co-transfected cells lacking light treatment (lane-2). Data shows that blue-light treatment promotes the formation of complexes between TCON and ECON.

Blue light induced association of TCON and ECON in the transiently co-transfected HeLa cells was investigated using FLIM-FRET experiments (Supplementary Materials and Methods) followed by a co-immunoprecipitation assay. The average fluorescence lifetime of EGFP fusion protein in cells expressing TCON alone was estimated to be 2.4 ns (Figure 2a). However, in the case of cells co-transfected with TCON and ECON, the average fluorescence lifetime of EGFP fusions reduced from 2.43 ns to 2.06 ns upon exposure to blue-light for 5 min at 8 mW/cm^2^ (Figure 2b). This significant reduction in fluorescence lifetime can be attributed to the FRET effect between EGFP and mCherry of the optically active fusion proteins due to the light-induced association/binding. Such interactions appear to be dependent both on time and intensity of illumination because of a similar magnitude of FRET response that was observed after 8 min, when the illumination power was reduced to 1 mW/cm^2^ (Supplementary Figure 3) [34]. However, a further 10-fold reduction in optical power to 0.01 mW/cm^2^ was unable to trigger any detectable interaction between the fusion proteins even after long-term exposure (Supplementary Figure 4). These data revealed a light-dependent association of TCON and ECON. This was further validated by co-immunoprecipitation (Co-IP) using EGFP antibody and analysis of the fractions by Western-Blot analysis using DNMT3A specific antibody. We have detected the ECON fusion protein (*ca.* 223 kDa) from the blue light exposed co-transfected cells (Figure 2c, lane-3). In contrast, no ECON fusion protein was immunoprecipitated from the co-transfected cells deprived of blue light exposure (Figure 2c, lane-2). Cells transfected with TCON served as the negative control, and did not show presence of DNMT3A (Figure 2c, lane-1). Collectively, the FLIM-FRET analysis, the Co-IP data, the studies shown in Figure 1, and the continued punctate staining pattern after exposure to blue-light show the formation of blue light dependent formation of TCON and ECON at telomeric and subtelomeric regions.

### Induced increase in methylation marks at the subtelomeric loci

**Figure 3:**
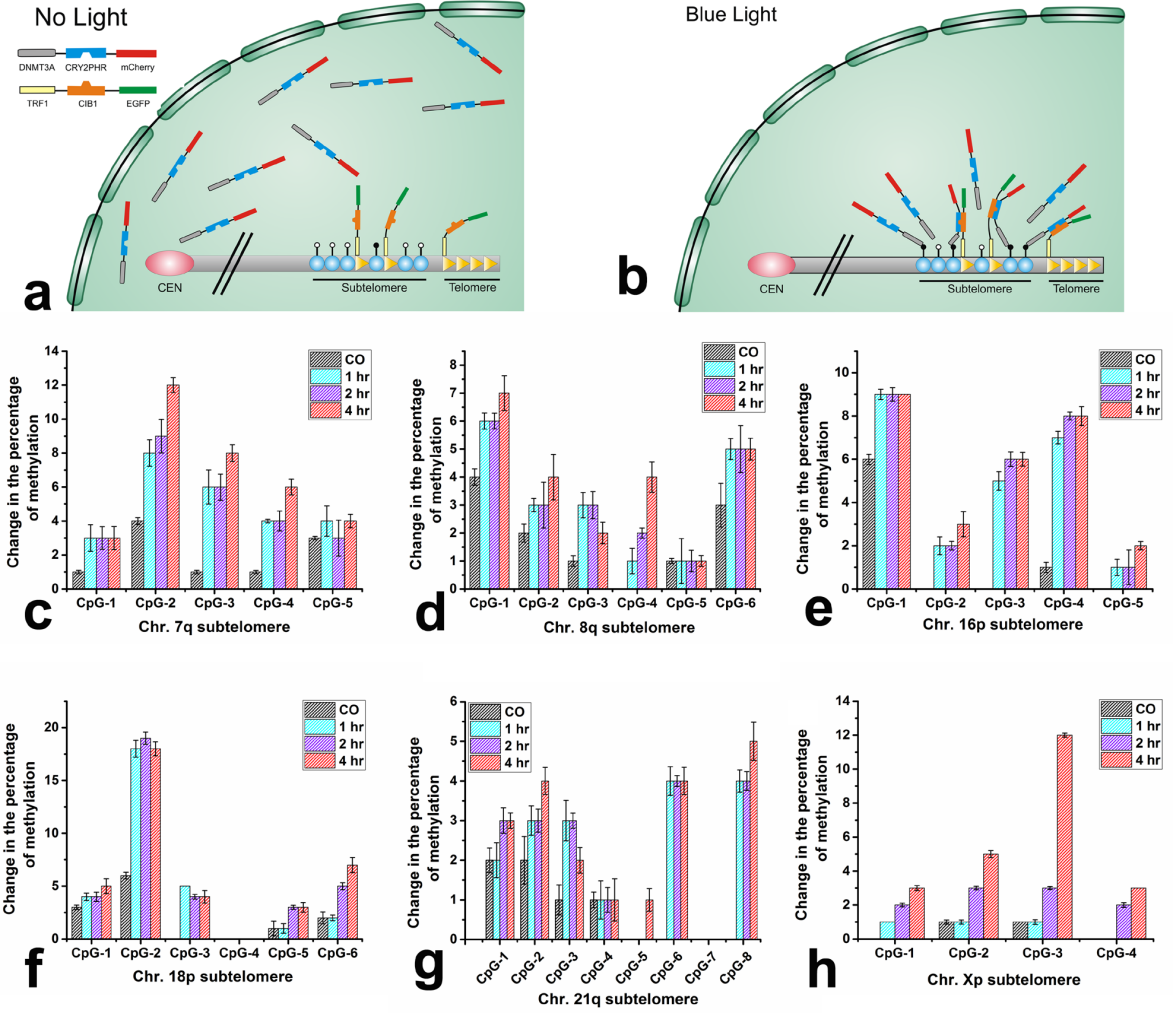
Blue light induced DNA-methylation at the subtelomeres Schematic showing that, ECON fusion proteins move freely within the nucleus in the absence of light **a.** however, their accumulation at the telomeric and subtelomeric sites is triggered by blue light driven association to the TCON fusion proteins bound to the ends of chromosome **b.** Analysis of changes in DNA methylation at selected subtelomeric CpG sites. The extent of changes in methylation was compared between co-transfected cells that were either untreated or illuminated for 1, 2, and 4 hr. Methylation levels were measured by pyrosequencing and expressed as % change at the subtelomeric region of chromosome 7q **c.** 8q **d.** 16p **e.** 18p **f.** 21q **g.** and Xp **h.** Varying levels of increase in methylation at distinct CpG loci was observed after blue light treatment for 1 hr, 2hr, or 4 hr. compared to the co-transfected (CO) cells without any illumination.

Initially, the ability to methylate genomic DNA by DNMT3A domain situated in the context of the ECON fusion was evaluated by measuring the global changes in methylation (Supplementary Figure 5). The overall methylation level changed from 1% in mock transfected cells to 1.6% in ECON transfected cells indicating catalytic activity of DNMT3A in ECON fusion. The schematic in Figure 3a and 3b illustrate the proposed changes leading to a local increase in concentration of ECON at a telomere and adjacent subtelomere due to the blue-light induced association with TCON bound to various loci of TTAGGG sequences at the chromosomal ends. We hypothesize that the localized ECON will increase the methylation at CpG loci in the subtelomeric region. The changes in methylation level at six different subtelomeric CpG sites of chromosomes (Chr.) were quantitatively determined after the cells were co-transfected and illuminated for different excitation time periods (1 hr, 2 hr, or 4 hr). The subtelomeric CpG sites of Chr. 7q, 8q, 16p, 18p, 21q, and Xp were selected due to their unique sequences, absence of telomere like repeats, lack of sequence gaps and varying distance of the CpG sites from the telomeres and interstitial TTAGGG sequences [35, 36]. The change in the methylation status at the subtelomeric CpG sites of six chromosomes (Chr.) in TCON and ECON expressing cells exposed to blue-light for the three experimented conditions was determined by pyrosequencing method. As expected, exposure to blue light caused varying levels of increase in methylation at the selected CpG sites (Figure 3c-3h) compared to the co-transfected cells without any light exposure. For example, we have observed highest level of changes in methylation at the following regions: 12% at the 2^nd^ CpG position of chr.7q (Figure 3c), 7% at the 1^st^ CpG position of chr.8q (Figure 3d), 9% at 1^st^ CpG position of Chr.16p (Figure 3e), 19% at 2^nd^ CpG position of Chr. 18p (Figure 3f), 5% at 8^th^ CpG position of Chr.21q (Figure 3g) and 12% at the 3^rd^ CpG position of chr.Xp (Figure 3h). The status of methylation at these subtelomeric CpG sites from the mock-transfected and illuminated cells are summarized in Supplementary Figure 6. Although the maximum changes in methylation were observed from the cells illuminated for 4 hr, the levels of increase were not dramatically different from the cells illuminated for 1 hr or 2 hr suggesting that significant methylation occurs during shorter duration exposures to blue-light.

Strikingly, CpG sites targeted by the optogenetic constructs were methylated only when they are located within 5 kb of the adjacent TTAGGG sequence. We did not observe any significant increase in the methylation level at two distal (> 5 kb) subtelomeric CpG sites of Chr. 21q and Chr. Xp, followed by co-transfection and illumination (Supplementary Figure 7b and 7c). Moreover, the change in methylation at off-target sites was evaluated at an additional region far from the subtelomeric sites by selecting the promoter of *HSPA13* gene located proximal to the centromere of chr.21 (Supplementary Figure 7a). Although random increase in methylation level was observed at certain CpG sites of these regions following co-transfection, no further increase in methylation was observed upon exposure to blue-light. This suggests that methylation at these sites might be independent of the association between TCON and ECON. Cumulative data also suggests that the methylation level at CpG sites, which are proximal (< 5 kb) to TTAGGG sites might be specifically subjected to the ECON derived DNMT3A activity.

### Effect of optically induced methylation on telomere length

**Figure 4:**
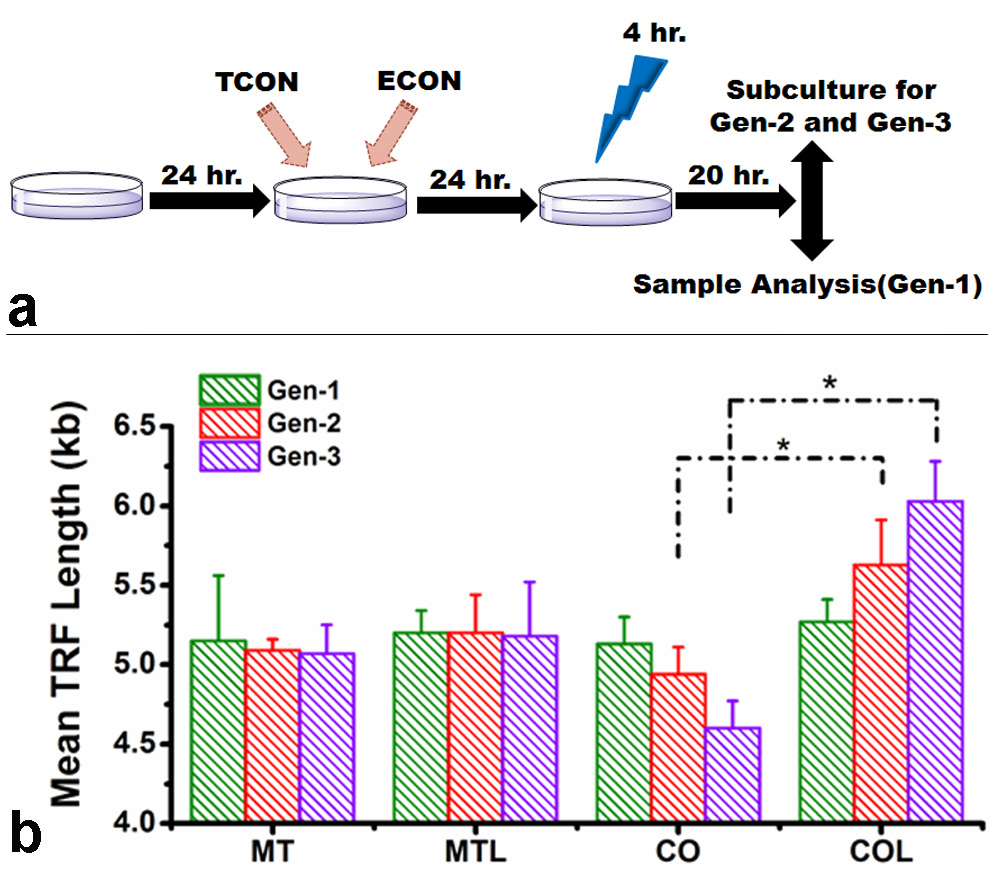
Change in telomere length and telomerase activity Schematic of the light-dark cycle, subjected to three generation of cells before telomere length measurement **a.** Telomere length was evaluated among the replicates of mock transfected (MT) and co-transfected (CO) cells both in absence or presence of blue light (L) (for 4 hr) over three consecutive generations; using Southern Blotting **b.** Treated cells were incubated for 24 hour of post-illumination, and subjected to telomere length measurements. Telomere length did not change significantly among mock transfected cells either in the presence or absence of light. Telomere length slightly decreased in co-transfected cells without illumination over generations. By contrast, a progressive increase in telomere length was observed for the blue light illuminated co-transfected cells. The change in telomere length between light treated and non-light treated CO cells was significant (*p* < 0.05) at the end of 2^nd^ and 3^rd^ generation of treatment.

The potential effect of light-induced subtelomeric methylation on telomere length was analyzed for three generations of Hela cells subjected to successive cycles of light-dark treatments as described in the Materials and Methods section (Figure 4a). The changes in mean telomere length (TRF) in treatment groups was determined with Southern Blot over three generations, compared to the initial non-treated cell population (Figure 4b; Supplementary Figure 8a). Insignificant changes in telomere length were observed in mock transfected cells either in the presence or absence of blue light. A slight decrease in telomere length was observed in co-transfected cells that were not exposed to blue light. The mean TRF length (kb) slightly reduced from 5.01 Kb in the Gen-1 to 4.9 Kb in the Gen-2 and 4.6 Kb in the Gen-3 samples. Surprisingly, a more dramatic yet opposite effect was observed in co-transfected cells exposed to blue-light, where the telomere length progressively increased for each generation (5.3 Kb for Gen-1, 5.7 Kb for Gen-2 and 6.03 Kb for Gen-3) (Figure 4b). Furthermore, we verified the increase in telomere length using quantitative PCR wherein a similar trend in the change in telomere repeats per reaction among the treated groups is observed (Supplementary Figure 8b). Moreover, we have evaluated the possible changes in telomere length in cells, treated individually with either TCON or ECON. We however, did not observe any significant changes in telomere length followed by the ECON treatment. In comparison, telomere lengths decreased gradually in cells, followed by the treatment with TCON (Supplementary Figure 9). Because HeLa cells are telomerase positive cancer cells, it is possible that enhanced subtelomeric methylation induced by blue light may have increased the access of telomerase to the telomeric DNA. Therefore, the telomerase activity was determined from cell extracts of the third generation using telomere repeat amplification (TRAP) assay (Supplementary Materials and Methods). Supplementary Figure 10 show that there were no significant changes relative telomerase activity in any of the cell extracts tested. Simultaneously, the potential cytotoxic effects of blue-light treatment and the co-transfection with TCON and ECON constructs were evaluated using a MTT based cell proliferation assay (Supplementary Figure 11). A 14% decrease in cell viability was observed when mock-transfected cells were exposed to blue light, whereas a nominal 3% increase was observed when light treated co-transfected cells were compared to mock-transfected cells with light exposure. Thus, both light treatment and co-transfection of TCON and ECON are likely to have relatively little effect on cell viability.

## DISCUSSION

Epigenetic modification via DNA methylation can cause significant effect on gene expression by exerting control over a variety of processes involved in cellular maintenance and proliferation [37]. However, artificially induced methylation at the selected sites on chromosomes presents an assortment of unique challenges. Here, by adopting optogenetic-epigenetic manipulation, we developed for the first time a tool to selectively increase the methylation status of the subtelomeric CpG islands. We demonstrate that site-directed methylation of subtelomeric regions has the potential to modify the global telomere length.

Two constructs were designed to demonstrate the light-directed site specific methylation. These constructs encode the TCON and the ECON fusion proteins with the latter acting as an effector due to the presence of DNMT3A. We selected DNMT3A over other DNA methyltransferases such as DNMT1 to create additional methylation because of its broader spectrum of activity. DNMT1 primarily maintains the methylation status at the hemimethylated CpG islands in the genome by specifically copying the methylation marks onto daughter strands created during replication [38, 39]. Capable of *de novo* methylation, DNMT3A creates methylation marks both at the unmethylated and hemimethylated CpG dinucleotides in addition to taking part in the maintenance of methylation patterns [40-42]. To preferentially localize DNMT3A to the subtelomeric regions it was fused to CRY2PHR, an optically active domain of CRY2 protein in ECON. CRY2PHR is capable of binding to its partner CIB1. The CRY2-CIB1 interactions are blue light dependent and reversible and have been used in a number of optogenetic systems [32, 33, 43]. The association of CRY2PHR and CIB1 is also rapid and their association kinetics has been characterized in our previous work [44].

In the targeting construct TCON, CIB1 exists as a fusion to the DNA binding domain from TRF1, which specifically binds to TTAGGG repeats found at telomeres and subtelomeres [22, 45]. We selected TRF1 to direct the optogenetic proteins selectively to each of the chromosome end, since it binds to double stranded telomeric DNA repeats that helped to anchor TCON fusion protein to the chromosome ends. The blue-light driven association between the CRY2PHR and CIB1 domains of ECON and TCON is a critical factor in localizing DNMT3A to the chromosome ends (Figure 3b). Using a variety of techniques, including single molecule fluorescence approaches, we demonstrated that the full-length fusion proteins localize to the nucleus wherein TCON presents a punctate nuclear staining pattern presumably at telomeres. FCS analysis showed low rates of diffusion of TCON as expected of a telomere binding protein bound to its target. Confirmatory evidence for TCON binding to telomeric DNA was obtained by isolation of TCON-DNA complexes by immunoprecipitation and analysis of bound DNA by qPCR. Typical punctate, overlapping staining pattern of TCON and TRF2, a telomeric binding marker, suggests that TRF1 of TCON and TRF2 are situated in close proximity, which strengthens the inference that TCON binds specifically to the telomeric repeat sequences. In addition, fluorescence lifetime analysis using FLIM-FRET and Co-IP data demonstrated the association between TCON and ECON occurs at the telomere and was dependent and sensitive to both time and intensity of blue light (Figure 2). Taken together, our data reveals the ability to spatiotemporally control the reversible assembly of the TCON-ECON complexes at the telomeres.

The demonstrated assembly of ECON-TCON complex at the telomeres opens the opportunity to localize DNMT3A at additional selective genome specific target sites. Although telomeric DNA is not a substrate for DNMT3A due to lack of CpG islands, the adjacent subtelomeric regions rich in CpG islands, was found to be an active target for selective DNA methylation. Co-transfection of TCON and ECON increased the methylation both globally and at the subtelomeric sites in the absence of blue light. For instance, the subtelomeric regions of interest and the selected control internal genomic region (the *HSPA13* gene situated near the centromere of chr.21) showed increased methylation as detected by pyrosequencing (Supplementary Figure 7b). The global increase in methylation detected in the absence of blue light is likely due to the overexpression of ECON from the CMV promoter and the presence of the native DNA binding domain of the intact DNMT3A of ECON which facilitates DNA binding and methylation of any CpG island. Interestingly, the level of methylation further increased only in the subtelomeric region but not in the interstitial genomic regions, when exposed to blue light and not in control regions. We evaluated the changes in methylation at six unique subtelomeric regions, which are variously distant from the TTAGGG repeat sequence. The tested unique regions in Chr. 18p and Chr. 21q were proximal (49 bp and 118 bp respectively), whereas in Chr. 16p, Chr. 8q, and Chr. Xp they were situated at a moderate distance from the TTAGGG sequence (206 bp, 774 bp, and 839 bp respectively) and in Chr. 7q the subtelomeric site was separated as far as 3.86 kb away from the TTAGGG repeat. The extent of blue light induced subtelomeric methylation varied between specific chromosomes as well as between CpG sites within individual chromosomes. We have also observed that the highest increment of methylation occurred at the CpG sites, when illuminated for a longer time period (4 hr), compared to the groups illuminated for comparatively a shorter period of time (2 hr or 1 hr). However, in some CpG sites the changes in methylation were not significantly different between the treated groups illuminated for different time-span. This phenomenon can be explained by the association kinetics between TCON and ECON. Although previous studies suggest that the interaction of CIB1 and CRY2 occurs within few seconds but their dissociation is much slower [32, 33]. The local concentration of the bound TCON and ECON proteins hence might be higher at the subtelomeric sites, which reduces the chance of unassociated (free) ECON proteins to assemble at the repeat sequences. The extent of optically-induced methylation at the subtelomeric sites, variously distant from the TTAGGG sequences is unlikely to be exclusively dependent upon the distance between the sequenced CpG regions and TTAGGG sites, but can be attributed to several factors. There might be a possibility that the DNMT effect could be more prominent at the hypomethylated regions unlike the hypermethylated subtelomeric regions. Additionally, due to the dynamic three-dimensional structure and the compaction of DNA, even adjacent CpGs may not have similar degree of accessibility to the DNMT. Although our study demonstrated changes in methylation at six subtelomeric sites, such changes are likely occurring at other CpG sites of the same chromosome as well as other chromosomes. We also show that the extent of methylation effects at the subtelomeric CpGs due to the assembly of TCON and ECON is dependent on the distance between the CpG sites and TTAGGG sequences. This ensures specificity of optogenetic targeting. Taken together, our study shows that by optogenetic targeting of DNMT3A to telomeres we can change the methylation level of subtelomeric CpG loci. Previously, TALE domain mediated targeting of optogenetic pairs to achieve histone modification and DNA demethylation at various gene promoters have been reported [32, 46]. To our knowledge this is the first report of selective modulation of the epigenetic status of subtelomeric regions.

Significantly, our studies show two divergent effects on telomere length in the presence of the optogenetic construct pairs in HeLa cells. We observe slight shortening of telomeres in TCON and ECON producing cells in the absence of blue light treatment. This reduction in telomeric length is consistent with previous studies showing overexpression of TRF1 that resulted in a gradual and progressive reduction in telomere length in tumor-cell line HT1080 and such an effect could be nullified using a dominant-negative allele of TRF1 without the telomeric DNA binding myb domain [14]. Analogous findings were reported for TRF2, a telomere binding protein associated with TRF1 [15]. Interestingly, the telomerase activity in both these cell lines remains unchanged and therefore inhibitory effects were attributed to cis-acting negative inhibition of telomere elongation [14].

Juxtaposed with the reduction in telomere length is an opposing effect caused by blue light treatment of co-transfected cells, namely the progressive increase in telomere length. Although, the increase in length of telomeres appears to be small (0.9 kb between Gen-1 to Gen-3), the increase is significant (*p* < 0.05) compared to co-transfected non-illuminated cells where a decrease in telomere length was observed best-possibly due to inhibitory effects from the overexpression of TCON fusion containing TRF1. We find the expression of ECON in co-transfected cells results in global increase in DNA methylation. Despite the global increase in methylation, a parallel expansion in telomere length was not observed in either individually or co-transfected cells lacking light treatment (Figure 4b and Supplementary Figure 8a). Therefore, the increase in telomere length after blue light treatment is likely to be directly related to the optically directed assembly of TCON and ECON at the telomeres and the abundant increase in methylation at subtelomeric regions. Furthermore, we find that the increase in subtelomeric methylation does not have any effect on telomerase activity in these cells. We propose that increased subtelomeric methylation can enhance the accessibility of telomerase to telomeric DNA either directly or indirectly through the regulation of positive and/or negative modulators of telomere length homeostasis.

The application of optogenetic tools to cell biological studies has allowed unprecedented spatiotemporal control in the manipulation of biological networks to extract mechanistic insights on cellular processes. To target specific DNA loci for epigenetic alterations, various DNA interaction domains such as TALE, zinc finger motifs have been integrated into the optogenetic tools [32, 46, 47]. We have expanded the optogenetic tool box by using CIB1-CRY2PHR to co-localize DNMT3A and TRF1 at the subtelomeric sites to alter the epigenetic state to study how epigenetic modulations regulate telomere length. Although the utility of our approach was demonstrated using the methytransferase DNMT3A, the modular design will permit substitution with variety of effectors such as other methyltransferases, demethylases, and histone modification enzymes to selectively manipulate the epigenetic status of chromosomal ends.

Epigenetic modifications are important in maintaining the heterochromatic state of telomeric and subtelomeric regions which in turn affects the telomere length and stability in both normal and cancerous cells [18, 20, 21, 25, 35, 48, 49]. Previously, a negative correlation between subtelomeric DNA methylation and the telomere length and telomeric recombination has been established in embryonic stem cells by experimental abrogation of DNMTs [20]. In contrast, positive correlation between subtelomeric methylation and telomere length was observed in a cohort of patients, diagnosed with Dyskeratosis congenital [27]. We show that selective increase in methylation (possibly through ECON) of subtelomeric CpG islands in TRF1 overexpressed cells increases the telomere length without an increase in telomerase activity. Our results support the previous finding that subtelomeric methylation might not alter the telomerase activity [49]. The observed increase in telomere length hence could be considered as a light induced synergistic effect from TCON and ECON and independent of telomerase activity. Biophysical tools such as optogenetics could be used to selectively perturb the epigenetic patterns at chromosomal ends to gain deeper mechanistic insights into the effect of epigenetic regulation on telomere biology of normal and cancer cells.

## MATERIALS AND METHODS

Detailed descriptions of all the materials and methods, mathematical derivations, and additional results are provided in the Supplementary Information.

### Design and construction of the optogenetic constructs

Optogenetic fusion proteins were generated by assembling the coding sequences of the desired proteins using standard restriction enzyme digest and ligation to the C-terminus of the HALO tag sequence (pHTNHaloTag CMV-neo Vector; Promega). EGFP (#23027 from A. LeBlanc laboratory), TRF1 (#16058 from T. D. Lange laboratory) of TCON and DNMT3A (#35521 from A. Riggs laboratory), CRY2-mCherry (#26866 from C. Tucker laboratory) of ECON were obtained from the Addgene plasmid repository (https://www.addgene.org/). The full CIB1 coding sequence including the flanking restriction sites for EGFP and TRF1 was synthesized with Genscript Inc. PCR conditions, PCR primers (Supplementary Table 1), sequencing primers (Supplementary Table 2) and full length sequence of both the constructs can be found in the Supplementary Information (Supplementary Sequences 1 and 2).

### Cell culture, transfection and microscopy

HeLa cells were seeded (0.7 × 10^6^) in petri-plates or culture flasks in the presence of DMEM/F-12 supplemented with 10% FBS, 1% glutamax and 1% penicillin-streptomycin (Life Technologies). Cells were subcultured five times after revival from the frozen stock, before actual experiments. Transient transfection was performed using Lipofectamine-LTX according to manufacturer's instructions. While imaging, cells were incubated with HBSS buffer (pH adjusted to 7.4 with 2 M NaOH) (Supplementary Materials and Methods).

### Co-immunostaining assay with TCON and TRF2

Post-transfected (24 hours after transfection) cells were fixed with 4% ice cold paraformaldehyde for 15 min, then permeated with 0.25% Triton X-100 for 30 min at room temperature. Blocking was performed with PBS solution containing 5% goat serum and 0.3% Triton X-100 for 1 h. Mouse monoclonal anti-TRF2 antibody (ab13579, Abcam) diluted 1:500 was incubated overnight with the cells at 4°C. After washing with PBS, goat anti-mouse secondary antibody conjugated with Alexa647 diluted 1:1000 was applied for 1 h at room temperature. Cells were completely rinsed with fresh PBS prior to imaging. Confocal imaging was performed with the PicoQuant system where a 465 nm laser was used to excite TRF1-GFP and a 633 nm laser was used to excite Alexa647.

### Single molecule fluorescence and illumination assays

The optogenetic association of ECON and TCON in the post-transfected live cells weremonitored with single-molecule fluorescence experiments, mostly FLIM-FRET and FCS (Supplementary Materials and Methods). In order to illuminate large populations of cells for the downstream biological assays, a mounted high-power LED with the peak wavelength at 455 nm (Thorlabs), controlled by a DC2100 LED driver, was applied. The illumination beam size was adjusted according to different surface areas of the culture vessels. Based on optimization, the illumination power for all of the subsequent experiments was determined to be 1-2 mW/cm^2^ for 60 min. At this intensity no cytotoxic effect were observed in our studies and is well below the threshold level and consistent with the past work on microscopy and optogenetics [32, 50].

### Co-immunoprecipitation coupled with western blot

Co-immunoprecipitation (Co-IP) was conducted with the Pierce Co-IP Kit (Thermo Fisher Scientific Inc.) as described in our previous study [39]. In brief, anti-EGFP monoclonal antibody (ab69314, Abcam) was immobilized using the AminoLink Plus coupling resin. 3 × 10^6^ cells transfected with TCON and ECON were lysed (25 mM Tris, 150 mM NaCl, 1 mM EDTA, 1% NP-40, 5% glycerol; pH 7.4) after illumination. About 1 mg of pre-cleared lysate was incubated overnight with the antibody-coupled resin at 4°C. After elution, the protein sample was analyzed by Western blotting. Western blot assay was performed as detailed in the Supplementary Materials and Methods, using an anti-DNMT3A antibody (ab23565, Abcam). The blot was developed with a secondary Ab from rabbit (ab150077; Thermo-Scientific). A 180 kDa pre-stained marker (26616; Life Technologies) was used as a reference for relative mass.

### BSP-PCR and pyrosequencing

The possible effect of light induced assembly of TCON and ECON on subtelomeric methylation was assessed with pyrosequencing. Changes in methylation level were demonstrated at six subtelomeric CpG sites from Chr. 7q, 8q, 16p, 18p, 21q, and Xp, which were located within 5 kb from the adjacent TTAGGG sequences. The changes in methylation level were also determined at the subtelomeric sites (at Chr. 21q and Xp), distal (>5 kb) to the adjacent TTAGGG sequences and at the promoter region of a centromere adjacent gene *HSPA13* at chr.21. These regions served as the negative control to the light induced methylation at the TTAGGG proximal subtelomeres. Details of the BSP and pyrosequencing assays can be found in Supplementary Materials and Methods, Results section. BSP-PCR, pyro-sequencing primers, PCR reaction conditions and sequences analyzed are summarized in the Supplementary Tables 3 and 4.

### Measurement of telomere length and telomerase activity

To evaluate the subtelomeric methylation effects on telomere length, cells expressing TCON and ECON were treated in light-dark cycle of 72 hr duration. Briefly, cells grown for 24 hr were co-transfected with TCON and ECON and allowed to grow for another 24 hr followed by a four-hour exposure to blue light. Subsequently, the cells were allowed to recover in the dark for 20 hours. Cells at the end of this typical cycle of treatment were referred to as generation-1 (Gen-1). Genomic DNA was extracted from cells of Gen-1 for determining telomere length. An aliquot of cells from Gen-1 were subjected to further rounds of light-dark cycle treatments as described to yield genomic DNA for generation-2 (Gen-2) and generation-3 (Gen-3). The DNA obtained from Gen-2and Gen-3, were actually at an interval of 144 hr. (6 days) and 216 hr. (9 days) respectively from the initial point of cell propagation which were considered the control (non-treated) cells. The telomere length was measured by Southern Blotting (Supplementary Materials and Methods), and analyzed by quantitative PCR analysis [51, 52] from three (n=3) independent rounds of experiments. The changes in telomere length at each generation were compared to the telomere length obtained from the initial non-treated control cells. Simultaneously we also evaluated the catalytic activity of telomerase with TRAP assay among the blue-light treated co-transfected cells from Gen-3 (Supplementary Materials and Methods).

### Statistical analyses

To determine the difference between average mean values between control and treated groups, we performed a two-tailed student's *t*-test. A *p*-value of < 0.05 was considered statistically significant in our analysis.

## SUPPLEMENTARY MATERIALS FIGURES


