# Supplementary material for: Optogenetic regulation of site-specific subtelomeric DNA-methylation
Source: Oncotarget. 2016 Jul 4;7(31):50380–91. doi: 10.18632/oncotarget.10394 (PMC5226589; doi:10.18632/oncotarget.10394)
Supplement: Supplementary file 1 [file oncotarget-07-50380-s001.pdf]

Optogenetic regulation of site-specific subtelomeric DNA-methylation

SUPPLEMENTARY DATA

SUPPLEMENTARY MATERIALS AND METHODS

Construction of the optogenetic fusion proteins

CloneAmpHiFi PCR Premix (639298, Clontech Laboratories Inc.) was used to amplify the inserts from the respective source plasmids. PCR was carried out as specified by the manufacturer for the template DNA concentration >100 ng with 35 cycles of amplification. PCR or restriction digested DNA fractions were gel purified (20021, QIAGEN). Purified inserts were then ligated and transformed into the stellar competent cells (PT5056-2, Clonetech Laboratories Inc.).

Western blot analysis

Individually and co-transfected cells were rinsed twice with ice-cold PBS followed by lysis with M-PER mammalian protein extraction reagent supplemented with Halt protease inhibitor cocktail (Thermo Scientific). The concentration of the extracted proteins was then determined with Coomassie Plus (Bradford) assay kit (Pierce), and the emission spectra of EGFP and mCherry in the cell free protein extract was confirmed by fluorescence spectrometry (Cary Eclipse, Agilent Technologies) (Supplementary Figure 1d, 1f, 1h). Fifteen micrograms of the extracted proteins were loaded per lane onto a 4% to 15% polyacrylamide gel (Bio-rad, USA) for electrophoresis and transferred to a nitrocellulose membrane (Biorad). The membrane was then blocked in 5% nonfat dry milk in (TBS-T) Tris-buffered-saline-Tween buffer (10mM Tris (pH 8.0), 140 mMNaCl, 0.1% Tween 20) overnight at 4°C, followed by incubation with mouse monoclonal anti-HALO Tag antibody (G9211; Promega, 1:1000 dilution) for 1 h in TBS-T. After washing three times with fresh TBS-T, the membrane was incubated with rabbit anti-mouse IgG (H+L) - AlexaFluor 680 conjugate antibody (A-21065; Life Technologies, 1:20,000 dilution) in the blocking buffer for 1h at room temperature. The membrane was re-washed with TBS-T for three times and scanned using a Li-Cor Odyssey scanner (CLx) system. A pre-stained SDS-PAGE standard (1610318; Biorad, USA) was used the marker to determine the size of the obtained proteins on blot.

Transfection method, fluorescence microscopy, FACS, and DAPI immunostaining

Plasmids encoding TCON and ECON constructs were extracted using Plasmid Midi kit (QIAGEN) and transfected the following day of seeding using Lipofectamine®LTX kit (Life Technologies, NY, USA) in low serum according to manufacturer's instruction. Briefly, 80% confluent cells were individually and co-transfected with 0.4 µg of TCON and 0.85 µg of ECON encoding plasmids in 35 mm petri-plates. 1 µl PLUS reagent, and 3 µl Lipofectamine LTX was used for lipofection. The quantity of the transfection reagents was multiplied according to the volume of the culture vessels. Post-transfected cells were grown for 24 h in the presence of 5% CO_2 at 37°C. Fluorescence microscopy (Olympus IX71 inverted microscope fitted with a mercury lamp) was primarily used to assess the expression of optogenetic constructs depending upon their respective fluorophore tags (EGFP or mCherry). Single/Co-transfected cells were also trypsinized (0.25% Trypsin-EDTA; Life Technologies, NY, USA), re-suspended in PBS and sorted with Fluorescence-assorted cell sorter (BD FACSAria™ III). Cells expressing individual/both EGFP and mCherry fluorophore were determined as a percentage of transfection efficiency of the constructs. Nuclear co-localization of the constructs was further validated via immunofluorescence after staining the co-transfected cells with DAPI (4', 6-Diamidino-2-Phenylindole). Briefly, 2% paraformaldehyde fixed cells were incubated in PBS for 15 min followed by staining with DAPI (300 nM). Stained cells were mounted with VECTASHIELD and observed under fluorescence microscope. Plasmid encoding pure EGFP (pCMV/myc/NUC/GFP; Life Technologies) was used as control in the chromatin immunoprecipitation assay and FCS measurements; 0.5 µg/35 mm petri-dish of NUC-GFP vector was used as input DNA during transfection.

Single-molecule based studies

HeLa cells were seeded into 35 mm petri dishes with the bottom thickness equal to No. 1 coverslip. Post-

transfected (24 hr) cells were visualized with a Microtime 200 scanning confocal time-resolved microscope system (Picoquant GmbH) fitted with a temperature control chamber to evaluate the intracellular behavior of ECON and TCON. A 465-nm picosecond pulsed diode laser was used to excite the EGFP tag. The excitation beam was delivered to the sample stage through an apochromatic 60 ×, 1.2 N.A. water immersion objective lens and the emitted fluorescence was collected with the same objective lens after which the emission was separated by a dual band dichroic mirror (z467/638rpc; Chroma). A 50-µm pinhole was applied to block the off-focus photons and the emission signal was further filtered by a band-pass filter (520 ± 20 nm, Chroma) before reaching the single photon avalanche photodiode detector (SPAD) (SPCM-AQR, PerkinElmer Inc.). The fluorescence signal was recorded using the TCSPC (time-correlated single-photon counting) module in the time-tagged time-resolved (TTTR) mode (TimeHarp200, Picoquant). Raw fluorescence lifetime images and autocorrelation functions were analyzed and exported by SymPhoTime software (PicoQuant) package for further processing. During the whole process, the laser power was precisely controlled by a Sepia PDL 808 driver, which has a modulating accuracy of 0.04 µW. For an input power of 0.5 µW, the actual average power density imposed on cells was measured to be about 1 mW/cm^2. In our optimization experiments, the power range from 0.02-8 mW/cm^2 for up to 10 min of illumination was used.

Fluorescence lifetime based förster resonance energy transfer (FLIM-FRET)

Fluorescence lifetime (τ) is defined as the average time the fluorophore stays at the excited state before emitting the first photon, and can be expressed as:

$$\tau = \frac{\Gamma}{\Gamma + k_{nr}}$$

In FLIM-FRET analysis, in order to visualize the change in fluorescence lifetime, a 150 × 150 pixelated image was first reconstructed from the pixel-by-pixel TCSPC information prior to determining FRET. For the time-domain measurement, the fluorescence lifetime can be obtained through fitting the TCSPC histogram when the frequency of the detected photon decays to 1/e of the highest level: $F(t) = F_0 e^{-t/\tau}$. FRET is a non-radiative energy transfer process and can be used to reflect the inter-molecule distance® below 10 nm, which is the spatial scale for most protein-protein interactions. In comparison with the intensity-based FRET measurement, FLIM-FRET is a more robust approach to sense the minute changes in the proximity between the donor and acceptor. Along with blue light illumination, it is expected that the optogenetic protein pair, CIB1 and CRY2, would isomerize inside the co-transfected cells. This event could be monitored by the FRET occurring in their individually fused fluorescent proteins, GFP and mCherry. A reduction in the lifetime of GFP is taken as an indicator of FLIM-FRET:

$$E_{FRET} = \frac{1}{1 + (\frac{r}{R_0})^6} = 1 - \frac{\tau_{DA}}{\tau_D}$$

R_0 is the Förster distance based on which the transferring efficiency (E_{FRET}) reaches 50% (~6 nm for EGFP-mCherry); τ_{DA} and τ_D are the lifetimes of GFP with and without the closely associated mCherry molecules, respectively.

Fluorescence correlation spectroscopy (FCS)

Autocorrelation function describes the similarity between observations as a function of the time lag (τ) between events. Within a femtoliter-level detection enabled by confocal microscopy setting, the fluorescence fluctuation caused by molecular diffusion can be interpreted by an autocorrelation function $G(\tau)$, mathematically giving the basis to calculate the average molecular number (N) and the diffusion time (τ_D):

$$G(\tau) = \frac{1}{N} \cdot (1 + \frac{\tau}{\tau_D})^{-1} \cdot (1 + \frac{1}{K^2} \cdot \frac{\tau}{\tau_D})^{\frac{1}{2}}$$

$\kappa = \frac{z_0}{w_0}$, describing the lateral and axial radii of the detection profile. For an intracellular protein with distinctive dynamic states such as the "free" and "bound", the single-component $G(\tau)$ can be expanded to a two-component model:

$$G(\tau) = \frac{1}{N} \cdot \left[(1-y) \cdot (1 + \frac{\tau}{\tau_D^{free}})^{-1} \cdot (1 + \frac{1}{\kappa^2} \cdot \frac{\tau}{\tau_D^{free}})^{-\frac{1}{2}} + y \cdot (1 + \frac{\tau}{\tau_D^{bound}})^{-1} \cdot (1 + \frac{1}{\kappa^2} \cdot \frac{\tau}{\tau_D^{bound}})^{-\frac{1}{2}}) \right]$$

y is the percentage of bound molecules. Based on this, the telomere binding efficiency of our constructed EGFP-CIB1-TRF1 (TCON) was evaluated.

Chromatin immunoprecipitation (ChIP)

HeLa cells were transfected with plasmids encoding TCON and pure GFP (pCMV/myc/nuc/GFP; Life Technologies) in parallel, as mentioned in Supplementary Materials and Methods. Preparation of the ChIP samples and purification of the target DNA were carried out using EZ-ChIP kit (17-371, EMD Millipore). Briefly, formaldehyde fixed cells were sonicated and the sheared chromatin was collected. Chromatin samples were then incubated with anti-EGFP immuno-precipitation (IP) antibody (ab290; Abcam) in parallel with the provided positive (Anti-RNA polymerase II) and negative (Normal mouse IgG) control antibodies by the manufacturer, for 12-16 h at 4°C. Isolated and purified IP-DNA samples were then amplified using PCR against the control or telomere specific primers reported elsewhere, and the product was visualized in 4 % agarose gel[3] (Supplementary Figure 2).

Determining the concentration of global methylation

Global change in the percentage of 5-methylcytosine genomic content in the co-transfected cells was determined with MethylFash™ Methylated DNA Quantification Kit (1034; Epigentek) according to manufacturer's protocol out of 100 ng genomic DNA, extracted with DNeasy Blood & Tissue kit (69504; Qiagen). Quantification of relative methylation of each treatment groups was determined as the percentage of 5mC in total DNA content (Supplementary Figure 5). All of the assays were performed in triplicate in comparison to the mock-transfected (treated with PLUS reagent and Lipofectamine LTX reagent) replicates.

Bisulfite PCR (BSP) and pyrosequencing

600 ng of genomic DNA, isolated from replicates of mock-transfected, and co-transfected (with and without light) cells were bisulfite converted with EZ DNA methylation kit (D5001; Zymo Research). PCR conditions and annealing temperature for the BSP-PCR was followed, as specified in the referred study (Supplementary Table 3). BSP-PCR was carried out using PyroMark PCR kit (978703; QIAGEN) per manufacturer's instruction. PCR products were then pyro-sequenced using PyroMark Q24 system (QIAGEN) supplemented with Pyromark Q24 advanced reagents (970922; QIAGEN) and against specific primers (Supplementary Table 4).

Measurement of telomere length with southern blotting

Average telomere lengths were determined following previously described method[1]. Briefly, the genomic DNA samples from 2 X 10^6 cells were digested with tetra-cutter restriction enzymes, HinfI, HaeIII and RsaI and resolved on a 0.8% agarose gel. In-gel hybridization analysis was performed with a ^{32}P-labeled $(TTAGGG)_4$ telomere probe and exposed to a phosphor screen (PhosphorImager; Molecular Dynamics, Sunnyvale, CA, USA). Mean telomere lengths were determined from PhosphorImager scans of gels hybridized using the Excel spreadsheet program TELORUN© as described earlier[2] (Supplementary Figure 8).

Measurement of telomere length with quantitative real-time PCR method (qPCR) method

HeLa cells were co-transfected and light exposed (Co-L) in parallel to TCON transfected and mock-transfected cells with light exposure (MTL) and without any treatment for three consecutive generations. The telomere length was then measured, out of the total extracted DNA samples against telomere specific primer set (Forward: CGGTTTGTTTGGGT TTGGGTTTGGGTTTGGGTTTGGGTT; Reverse: GGCTTGCCTTACCCTTACCCTTACCCTTACC CTTACCCT) using a qPCR method[3] with a minor modification. Briefly, six different dilutions (500, 250, 100, 50, 5, 0.5 picogram respectively) of a telomere oligomer standard (TTAGGG repeated for 14 times; IDT) was used to establish a standard curve by plotting the amount of telomere sequence (in the X-axis) against their corresponding cycle threshold (C_T) values (Supplementary Figure 9). Instead of PBR3222, we have used PUC-19 plasmid to retain the uniform DNA concentration (20 ng) of the PCR reaction. The cycle threshold (C_T) values from each tube containing equal concentration of DNA but of different telomere length were then fitted to the telomere standard curve to determine the length of telomere [log (kb)] / reaction. However, we have not adjusted the telomere lengths in respect to any single copy genes such as 36B4. This gives us a real flavor of telomere repeat addition per reaction of the experiment.

Determining telomerase activity with TRAP assay

Telomerase activity assays were performed on 3-[(3-cholamidopropyl) dimethylammonio]-1-propanesulfonate (CHAPS) extracts containing 0.5 µg of the total protein using the TRAPeze telomerase detection kit (Millipore). The reaction products were resolved by electrophoresis in 12.5% polyacrylamide gels and visualized by SYBR Green phosphorimaging as previously described[4,5] (Supplementary Figure 10). The commercial telomerase positive cells (Millipore) was used as a positive control and heat inactivated

telomerase positive cell extract was used as a negative control.

Determining cell proliferation with MTT assay

MTT (3-(4, 5-dimethylthiazol-2-yl)-2, 5-diphenyl-2*H*-tetrazolium bromide) based cell proliferation assay was performed to assess the possible effect of telomere shortening on cellular proliferation. Cells were individually transfected with TCON in comparison to the co-transfected-light exposed and mock transfected cells with and without the exposure of blue light for three consecutive generations (similar to the telomere length measurement assay). Since, the highest level of reduction in telomere length was obtained after the 3rd generation of treatment, MTT assay was performed for the cell-group of 3rd generation. The MTT assay was performed following an available standard protocol with required modifications[6]. Briefly, post transfected (in T-25 flasks) cells were light exposed as mentioned earlier and re-seeded into 96-well plates to further incubate for 48 hr. Cells were then treated with MTT reagent (20 µl of 5 mg/mL) and incubated for 4 hr at 37°C in the presence of CO_2. The supernatant was replaced with DMSO and the optical density was recorded on a microplate reader (Spectra max plus 384, Molecular Devices, USA) at 490 nm to determine the percentage of cell viability (Supplementary Figure 11).

SUPPLEMENTARY RESULTS

Supplementary Note 1

The pyrogram data revealed that all of the non CpG cytosines (C) sites were displayed as thymines (T), which implied that the bisulfite-induced conversion of unmethylated cytosine to uracil was complete in our DNA samples. This serves as the basis for subsequent calculation of methylation rates by measuring the relative peak heights of C and T peaks at each CpG in the sequencing traces.

Supplementary Note 2

Purity and concentration of DNA and RNA samples at each step of the performed assays were determined at 260/280 using a nanodrop spectrometer (Nanodrop ND-1000, Thermo Scientific) followed by agarose gel electrophoresis (for DNA only). All of the DNA primers were obtained from the Integrated DNA technologies (IDT), USA. All of the common chemicals unless specified were purchased from Sigma Aldrich (MO, USA). DNase/RNase free water (10977-015, Life Technologies) was used throughout for experiments.

Supplementary Note 3

As presented in Supplementary Figure 1, co-transfection rates of both TCON and ECON were relatively high at 70%. As a result, we would expect that about 30% of the populations would not respond to blue light and would not reflect alteration in telomere length. Therefore, we would expect a bi-or multi-modal distribution of telomere length in population treated with blue light as observed qualitatively via telomere length distributions in Southern blot analysis.

SUPPLEMENTARY REFERENCES

1. Huda, N., Tanaka, H., Herbert, B.S., Reed,.T, Gilley, D. Shared environmental factors associated with telomere length maintenance in elderly male twins. Aging Cell 6, 709-13 (2007).
2. Herbert, B.S., Shay, J.W., Wright, W.E. Analysis of telomeres and telomerase. Curr. Protoc. Cell Biol. 18, 18.6 (2003).
3. O'Callaghan, N.J., Dhillon, V.S., Thomas, P., Fenech, M. A quantitative real-time PCR method for absolute telomere length. Biotechniques 44, 807-809 (2008).
4. Herbert, B.S., Hochreiter, A.E., Wright, W.E., Shay, J.W. Nonradioactive detection of telomerase activity using the telomeric repeat amplification protocol. Nat.Protoc. 1, 1583-90 (2006).
5. Kannan, N., Huda, N., Tu, L., Droumeva, R., Aubert, G., Chavez, E., Brinkman, R.R., Lansdorp, P., Emerman, J., Abe, S., Eaves, C., Gilley, D. The luminal progenitor compartment of the normal human mammary gland constitutes a unique site of telomere dysfunction. Stem. Cell. Rep. 1, 28-37(2013).
6. Twentyman, P.R. & Luscombe M. A.Study of some variables in tetrazolium dye (MTT) based assay for cell growth and chemosensitivity. Brit. J. Cancer. 56, 379-285(1987).

Supplementary Sequence 1: Amino acid sequences of the HALO TAG—EGFP—linker—CIB1--NLS—linker---TRF1 (~149 KD) construct.

MAEIGTGFPFDPHYVEVLGERMHYVDVGPRDGTPVLFLHGNPTSSYVWRNIIPHVAPTHRCIAPDLIGMGK SDKPDLGYFFDDHVRFMDAFIEALGLEEVVLVIHDWGSALGFHWAKRNPERVKGIAFMEFIRPIPTWDEWPEFAR ETFQAFRTTDVGRKLIIDQNVFIEGTLPMGVVRPLTEVEMDHYREPFLNPVDREPLWRFPNELPIAGEPANIVALVE EYMDWLHQSPVPKLLFWGTPGVLIPPAEAARLAKSLPNCKAVDIGPGLNLLQEDNPDLIGSEIARWLSTLEISGE-PTTEDLYFQSDNAIASEF

MVSKGEELFTGVVPILVELDGDVNGHKFSVSGEGEGDATYGKLTLKFICTTGKLPVPWPTLVTTLTYGVQCFS RYPDHMKQHDFFKSAMPEGYVQERTIFFKDDGNYKTRAEVKFEGDTLVNRIELKGIDFKEDGNILGHKLEYNYNS HNVYIMADKQKNGIKVNFKIRHNIEDGSVQLADHYQQNTPIGDGPVLLPDNHYLSTQSALSKDPNEKRDHMVLLE FVTAAGITLGMDELYK

ALESGSTPA

MNGAIGGDLLLNFPDMSVLERQRAHLKYLNPTFDSPLAGFFADSSMITGGEMDSYLSTAGLNLPMMYGETT VEGDSRLSISPETTLGTGNFKKRKFDTETKDCNEKKKKMTMNRDDLVEEGEEEKSKITEQNNGSTKSIKKMKHKA KKEENNFSNDSSKVTKELEKTDYIHVRARRGQATDSHSIAERVRREKISERMKFLQDLVPGCDKITGKAGMLDEIIN YVQSLQRQIEFLSMKLAIVNPRPDFDMDDIFAKEVASTPMTVVPSPEMVLSGYSHEMVHSGYSSEMVNSGYLHVN PMQQVNTSSDPLSCFNNGEAPSMWDSHVQNLYGNLGVGTSVPAG

SPKKKRKVEAS

GRDADPTEEQ

MAETERNDEEQFECQELLECQVQVGAPEEEEEEEEDAGLVAEAEAVAAGWMLDFLCLSLCRAFRDGRSEDF RRTRNSAEAIIHGLSSLTACQLRTIYICQFLTRIAAGKTLDAQFENDERITPLESALMIWGSIEKEHDKLHEEIQNLIKI QAIAVCMENGNFKEAEEVFERIFGDPNSHMPFKSKLLMIISQKDTFHSFFQHFSYNHMMEKIKSYVNYVLSEKSSTF LMKAAAKVVESKRTRTITSQDKPSGNDVEMETEANLDTRKRSHKNLFLSKLQHGTQQQDLNKKERRVGTPQSTK KKKESRRATESRIPVSKSQPVTPEKHRARKRQAWLWEEDKNLRSGVRKYGEGNWSKILLHYKFNNRTSVMLKDR WRTMKKLKLISSDSED*

Supplementary Sequence 2: Amino acid sequences of the HALO TAG—DNMT3A—linker—CRY2-mCherry (~223 KD) construct.

MAEIGTGFPFDPHYVEVLGERMHYVDVGPRDGTPVLFLHGNPTSSYVWRNIIPHVAPTHRCIAPDLIGMGK
SDKPDLGYFFDDHVRFMDAFIEALGLEEVVLVIHDWGSALGFHWAKRNPERVKGIAFMEFIRPIPTWDEWPEFAR
ETFQAFRTTDVGRKLIIDQNVFIEGTLPMGVVRPLTEVEMDHYREPFLNPVDREPLWRFPNELPIAGEPANIVALVE
EYMDWLHQSPVPKLLFWGTPGVLIPPAEAARLAKSLPNCKAVDIGPGLNLLQEDNPDLIGSEIARWLSTLEISGE-
PTTEDLYFQSDNAIASEFGSANTGA

MPAMPSSGPGDTSSSAAEREEDRKDGEEQEEPRGKEERQEPSTTARKVGRPGRKRKHPPVESGDTPKDPAVI
SKSPSMAQDSGASELLPNGDLEKRSEPQPEEGSPAGGQKGGAPAEGEGAAETLPEASRAVENGCCTPKEGRGAPAE
AGKEQKETNIESMKMEGSRGRLRGGLGWESSLRQRPMPRLTFQAGDPYYISKRKRDEWLARWKREAEKKAKVIA
GMNAVEENQGPGESQKVEEASPPAVQQPTDPASPTVATTPEPVGSDAGDKNATKAGDDEPEYEDGRGFGIGELVW
GKLRGFSWWPGRIVSWWMTGRSRAAEGTRWVMWFGDGKFSVVCVEKLMPLSSFCSAFHQATYNKQPMYRKAI
YEVLQVASSRAGKLFPVCHDSDESDTAKAVEVQNKPMIEWALGGFQPSGPKGLEPPEEEKNPYKEVYTDMWVEPE
AAAYAPPPPAKKPRKSTAEKPKVKEIIDERTRERLVYEVRQKCRNIEDICISCGSLNVTLEHPLFVGGMCQNCKNCF
LECAYQYDDDGYQSYCTICCGGREVLMCGNNNCCRCFCVECVDLLVGPGAAQAAIKEDPWNCYMCGHKGTYGL
LRRREDWPSRLQMFFANNHDQEFDPPKVYPPVPAEKRKPIRVLSLFDGIATGLLVLKDLGIQVDRYIASEVCEDSITV
GMVRHQGKIMYVGDVRSVTQKHIQEWGPFDLVIGGSPCNDLSIVNPARKGLYEGTGRLFFEFYRLLHDARPKEGD
DRPFFWLFENVVAMGVSDKRDISRFLESNPVMIDAKEVSAAHRARYFWGNLPGMNRPLASTVNDKLELQECLEH
GRIAKFSKVRTITTRSNSIKQGKDQHFPVFMNEKEDILWCTEMERVFGFPVHYTDVSNMSRLARQRLLGRSWSVPV
IRHLFAPLKEYFACV

GTSGSLESGSTP

AMKMDKKTIVWFRAMKMDKKTIVWFRRDLRIEDNPALAAAAHEGSVFPVFIWCPEEEGQFYPGRASRWWM
KQSLAHLSQSLKALGSDLTLIKTHNTISAILDCIRVTGATKVVFNHLYDPVSLVRDHTVKEKLVERGISVQSYNGDLL
YEPWEIYCEKGKPFTSFNSYWKKCLDMSIESVMLPPPWRLMPITAAAEAIWACSIEELGLENEAEKPSNALLTRAWS
PGWSNADKLLNEFIEKQLIDYAKNSKKVVGNSTSLLSPYLHFGEISVRHVFQCARMKQIIWARDKNSEGEESADLFL
RGIGLREYSRYICFNFPFTHEQSLLSHLRFFPWDADVDKFKAWRQGRTGYPLVDAGMRELWATGWMHNRIRVIVSSF
AVKFLLLPWKWGMKYFWDTLLDADLECDILGWQYISGSIPDGHELDRLDNPALQGAKYDPEGEYIRQWLPELARL
PTEWIHHPWDAPLTVLKASGVELGTNYAKPIVDIDTARELLAKAISRTREAQIMIGAAARDPPVATMVSKGEEDNMA
IIKEFMRFKVHMEGSVNGHEFEIEGEGEGRPYEGTQTAKLKVTKGGPLPFAWDILSPQFMYGSKAYVKHPADIPDYL
KLSFPEGFKWERVMNFEDGGVVTVTQDSSLQDGEFIYKVKLRGTNFPSDGPVMQKKTMGWEASSERMYPEDGAL
KGEIKQRLKLKDGGHYDAEVKTTYKAKKPVQLPGAYNVNIKLDITSHNEDYTIVEQYERAEGRHSTGGMDELYK*

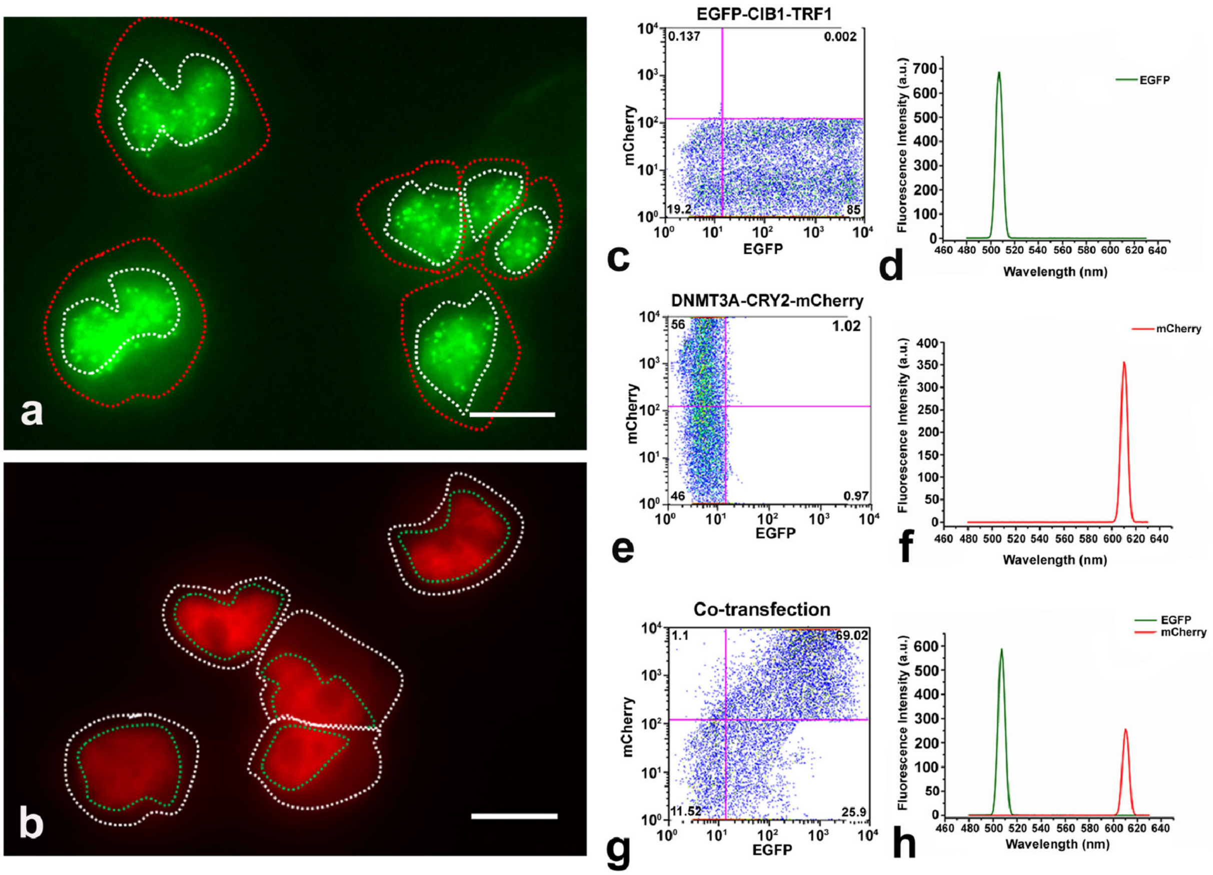

Supplementary Figure S1: Analysis of transfection efficiency of the optogenetic constructs TCON and ECON. Transient expression of TCON and ECON results in nuclear localization in HeLa cells with a characteristic punctate pattern for TCON **a.** whereas ECON **b.** showed diffused distribution. Dotted lines demarcate cytoplasmic and nuclear compartments (scale bar 20 µm). Fluorescence assorted cell-sorting analysis revealed that the average transfection efficiency was 85% for TCON **c.**, 56% for ECON **e.**, and 69% for TCON plus ECON **g.** in HeLa cells. The total protein was extracted from both the individually and co-transfected cells, and the emission spectra of the fluorescent tags (EGFP and mCherry) in the fusion proteins TCON **d.**, ECON **f.** and TCON plus ECON **h.** were recorded using a fluorimeter. This assay also showed the integrity of the fusion proteins in cell free extracts.

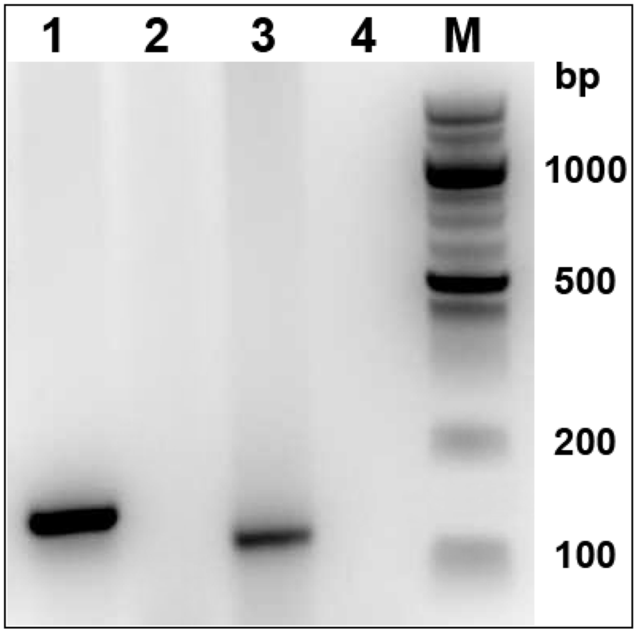

Supplementary Figure S2: Extracts of cells expressing TCON and control EGFP were immunoprecipitated using the anti-EGFP antibody and the co-purifying DNA fraction was used as a template in PCR with telomere specific primers. The amplicons in lane-3 and their absence in lane-4 indicate that telomeric DNA was pulled down by the EGFP antibody from the TCON transfected cells but not from the EGFP expressing cells. DNA fractions pulled down by anti-RNA polymerase II antibody was used as positive control (lane-1) and normal mouse IgG was used as negative control (lane-2). The observed telomere specific amplification in the PCR end-product suggests the binding of TCON to the telomeres.

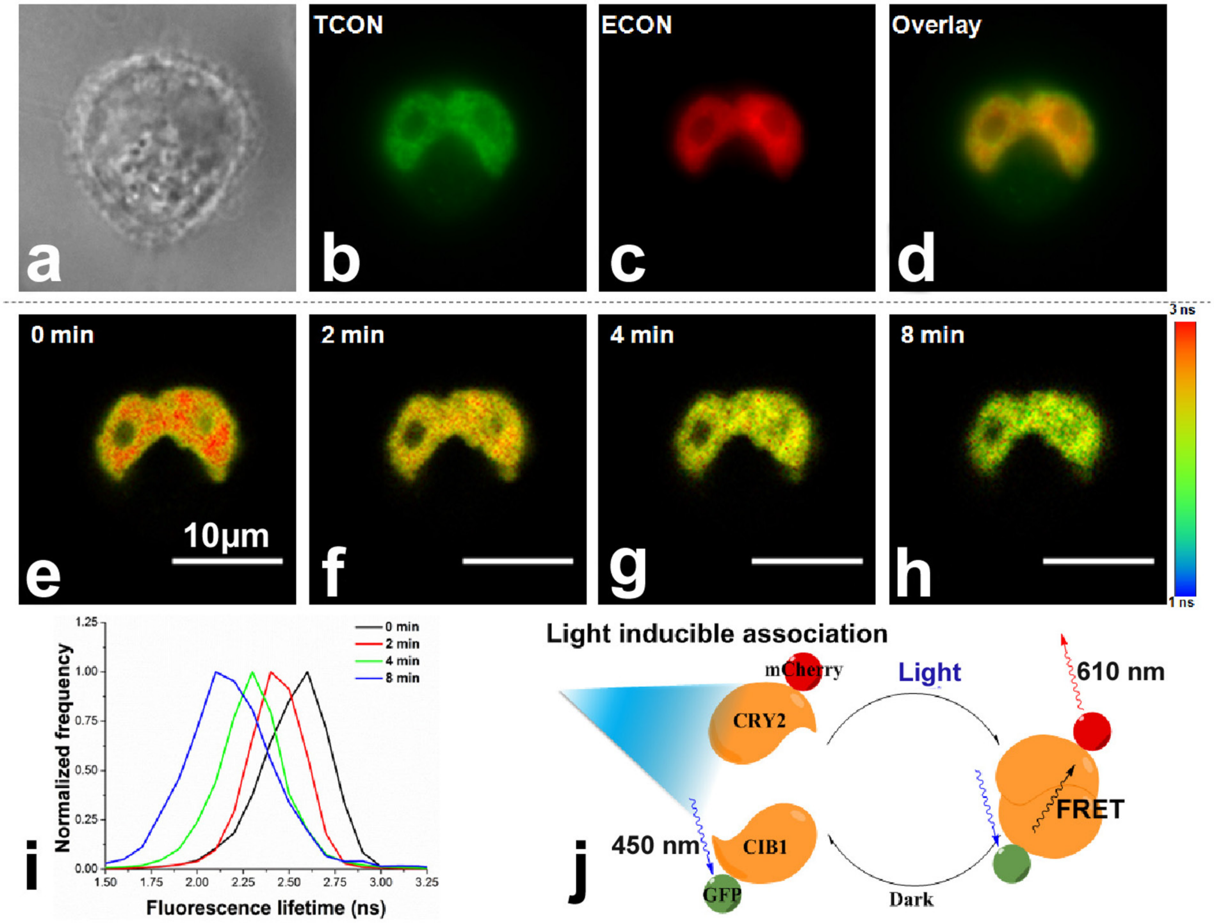

Supplementary Figure S3: Blue light induced interaction of optogenetic fusion proteins. Bright field **a.** and fluorescence images of the TCON **b.** and ECON **c.** fusion proteins along with their overlay **d.** of a representative HeLa cell transiently co-transfected with TCON and ECON constructs. Time-lapse images of the same cell after illuminated at an intensity of 1 mW/cm^2 for the indicated time periods **e-h.** Change in fluorescence lifetime as a function of time of exposure to blue-light indicates light induced association of ECON and TCON fusion proteins **i.** Bright field images of the same cells were used as for background normalization. Graphical illustration of FLIM-FRET induced by the association of ECON and TCON fusions upon exposure to blue light **j.**

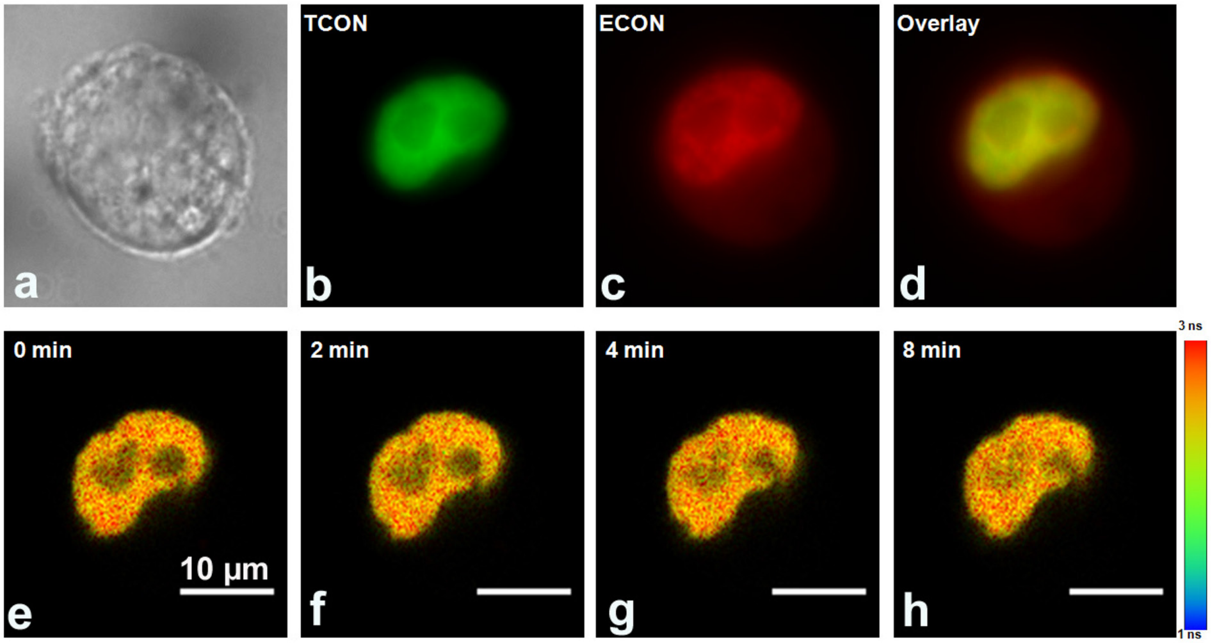

Supplementary Figure S4: Effect of the intensity of light on the association of optogenetic fusion proteins TCON and ECON. Images of representative HeLa cells co-transfected with TCON and ECON using the bright field **a.** and fluorescence optics, specific for TCON **b.** and ECON **c.** fusion proteins. The overlay of ECON and TCON images are shown **d.**Time lapse **e-h.** images of the same cell after exposure to blue-light at an illumination power of 0.01 mW/cm^2 for the indicated time periods. Bright field images of the same cells were used for background normalization. The lack of significant FLIM-FRET signal between panel e and h (compared to panel e and h of Supplementary Figure 3, exposed to 10-fold higher blue-light intensity) suggested that the low-intensity of illumination does not lead to noticeable association between TCON and ECON.

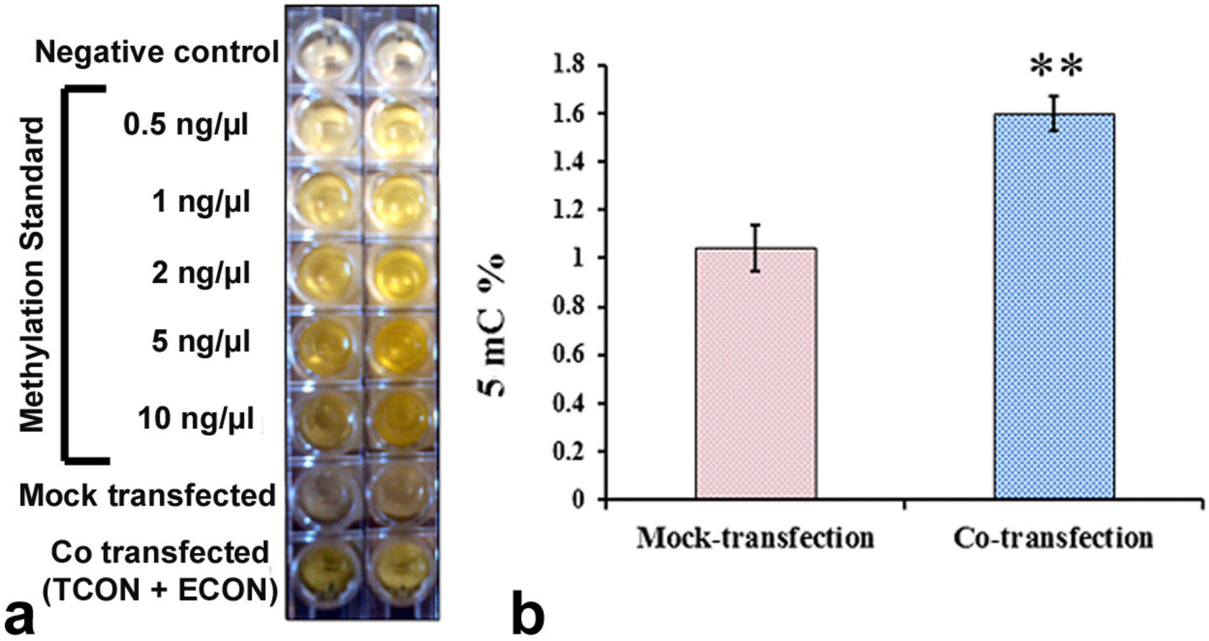

Supplementary Figure S5: Quantification the percentage of 5 methylcytosine (5mC). The percentage of 5mC in the co-transfected (with ECON and TCON) cells were found to be significantly higher in comparison to the mock transfected cells **b.** The left panel **a.** represents a comparative optical density of mock and co-transfected replica compared to the methylated standard (0.5 ng/µl to 10 ng/µl), provided by the manufacturer. Results correspond to the mean and S.D. of three independent measurements (n=3; **$p < 0.05$).

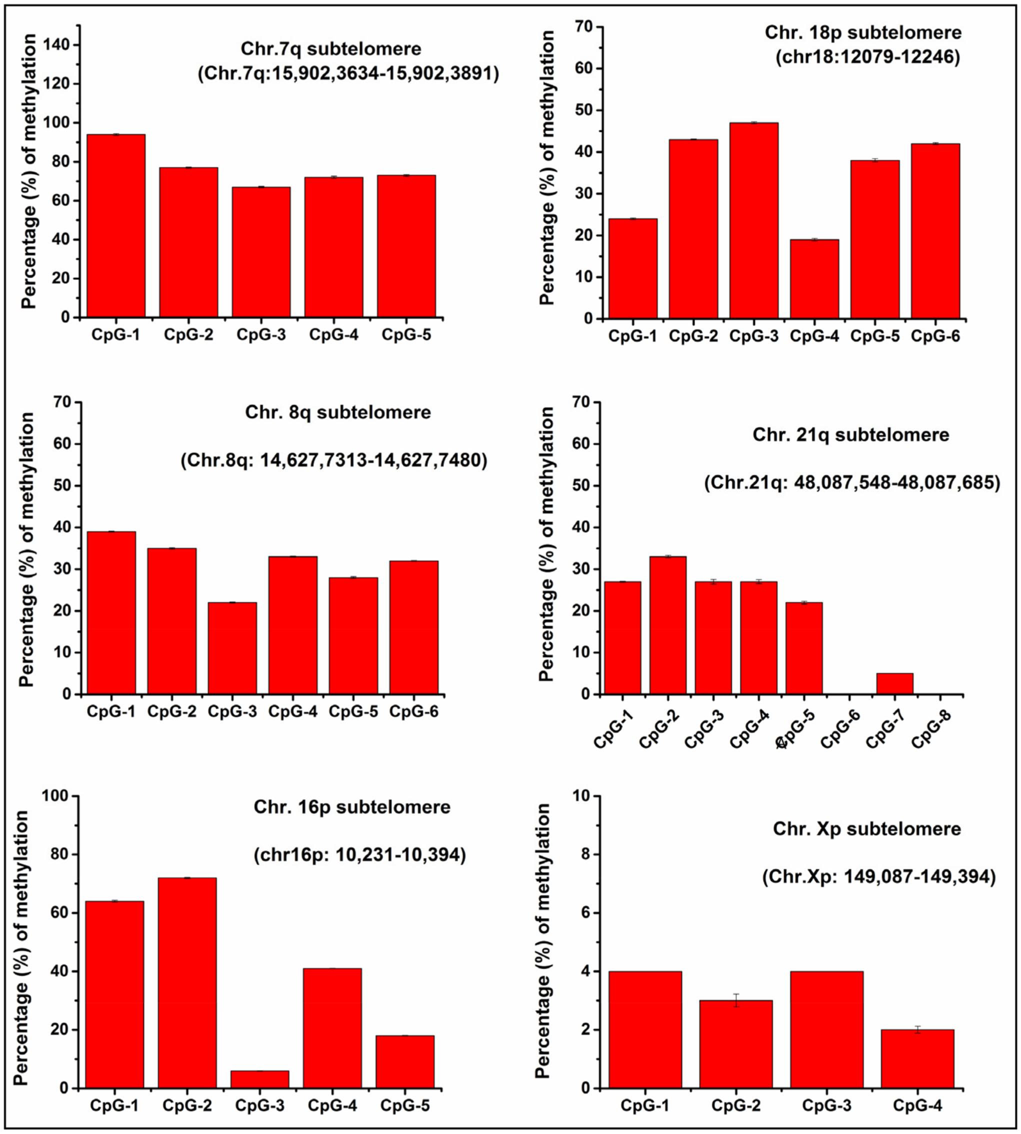

Supplementary Figure S6: Analysis of methylation at selected subtelomeric loci in mock-transfected illuminated cells. The percentage (%) of methylation at selected CpG sites of the target subtelomeric regions was determined with pyrosequencing analysis. The bars represent methylation density at the CpG sites as obtained from the HeLa genomic DNA from mock-transfected with light treatment. These values were normalized to determine the change in percentage (%) of methylation after each treatment in the present study.

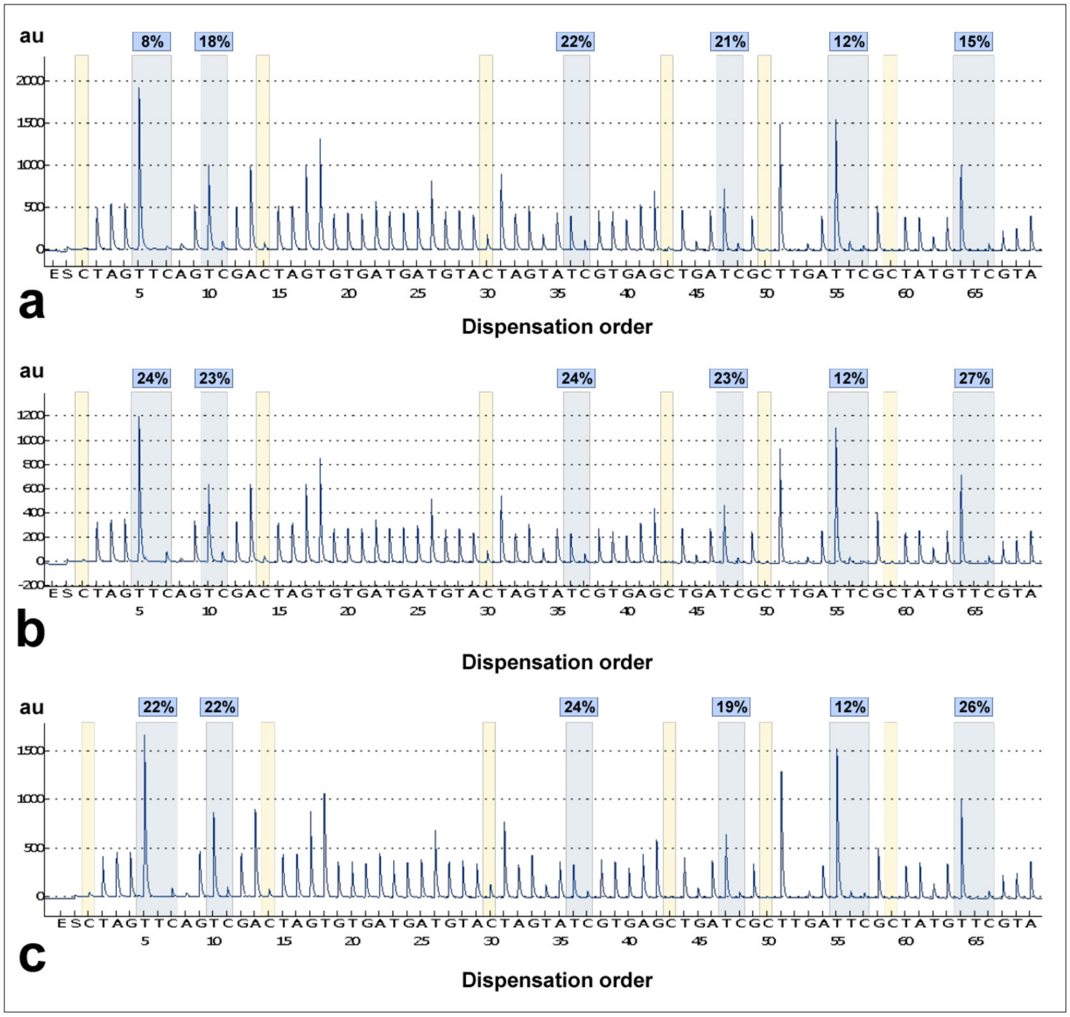

Supplementary Figure S7a: The change in methylation level at a sequenced region (15,755,381-15,755,581) of the HSPA13 gene promoter. The changes in methylation level were quantitatively determined by pyrosequencing at six CpG sites on the HSPA13 gene promoter, adjacent to the centromeric region of chr.21. The y-axis stands for the signal intensity in arbitrary units (a.u.), while x-axis stands for nucleotide dispensation order. The methylation level at each CpG site of the sequenced region was observed among the mock-transfected **a.**, co-transfected; non-illuminated **b.**, and co-transfected; blue light exposed **c.** cells. The methylation level at CpG-1, CpG-2 and CpG-6 were noticeably increased among the co-transfected non illuminated cells (b) compared to the mock-transfected cells (a). The increase in methylation level can be logically attributed to the catalytic activity of DNMT3A in the ECON fusion protein. However, we did not observe any further increase in the methylation level after blue-light illumination, compared to the non-illuminated co-transfected cells (c). This indicates that the change in methylation level at this region is independent of the blue-light triggered association of TCON and ECON.

(*Continued*)

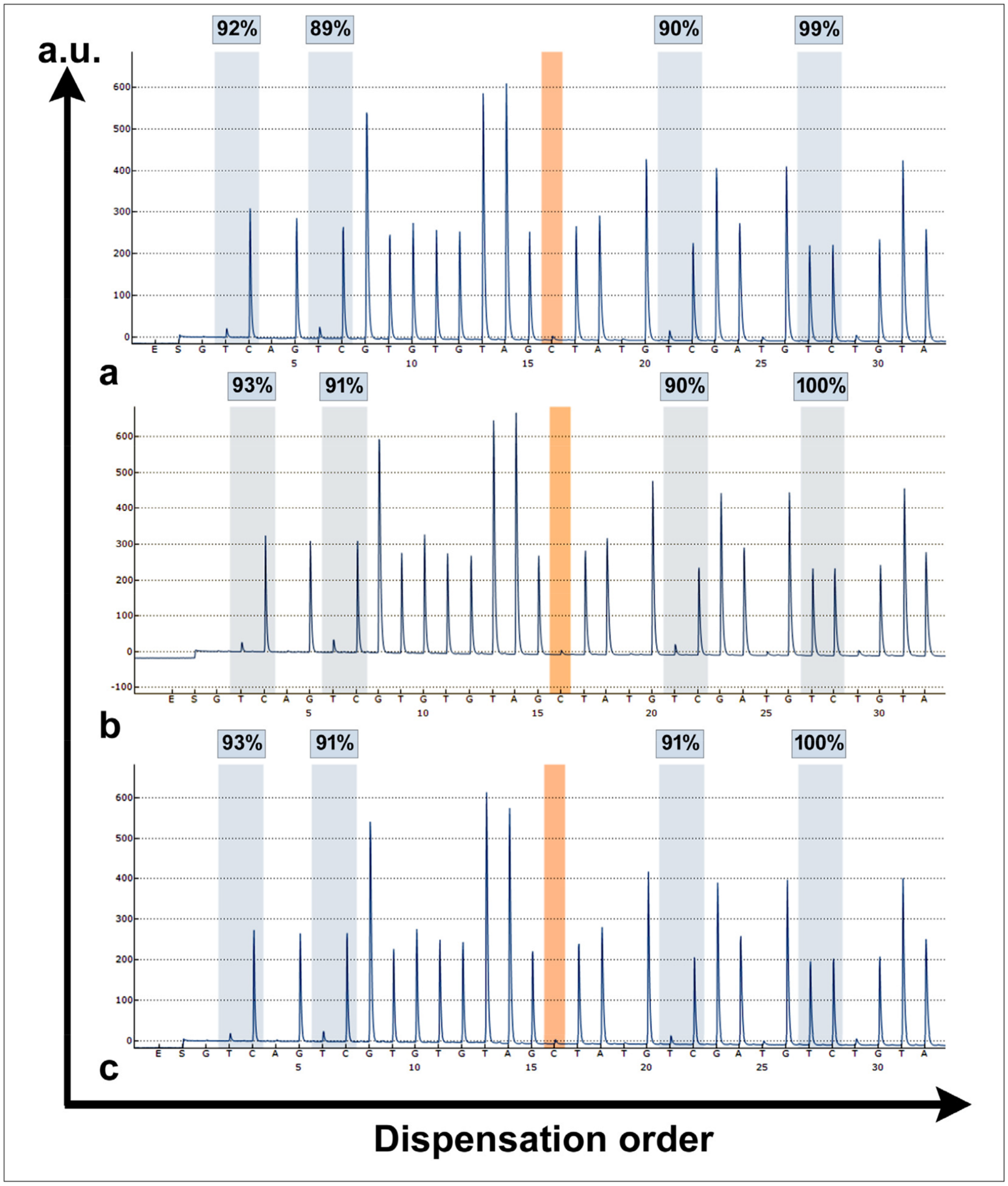

Supplementary Figure S7b: The change in methylation level at a distant (> 5 kb) subtelomeric region in Chr. 21q. The changes in methylation level were quantitatively determined by pyrosequencing at four CpG sites at a randomly chosen distant subtelomeric region in Chr. 21 (Chr.21:47,817,313-47,817,340). The sequenced site is 9.6 kb distant from the adjacent TTAGGG site. The y-axis denotes signal intensity in arbitrary units (a.u.), while x-axis denote nucleotide dispensation order. The methylation level at each CpG site of the sequenced region was observed among the mock-transfected-illuminated **a.**, co-transfected; non-illuminated **b.**, and co-transfected; blue light exposed **c.** cells. The highest increase in methylation was only 2% at the CpG-2 of the co-transfected cells with or without light treatment. We also did not observe significant increase in methylation level at any of the remaining CpG sites.

(*Continued*)

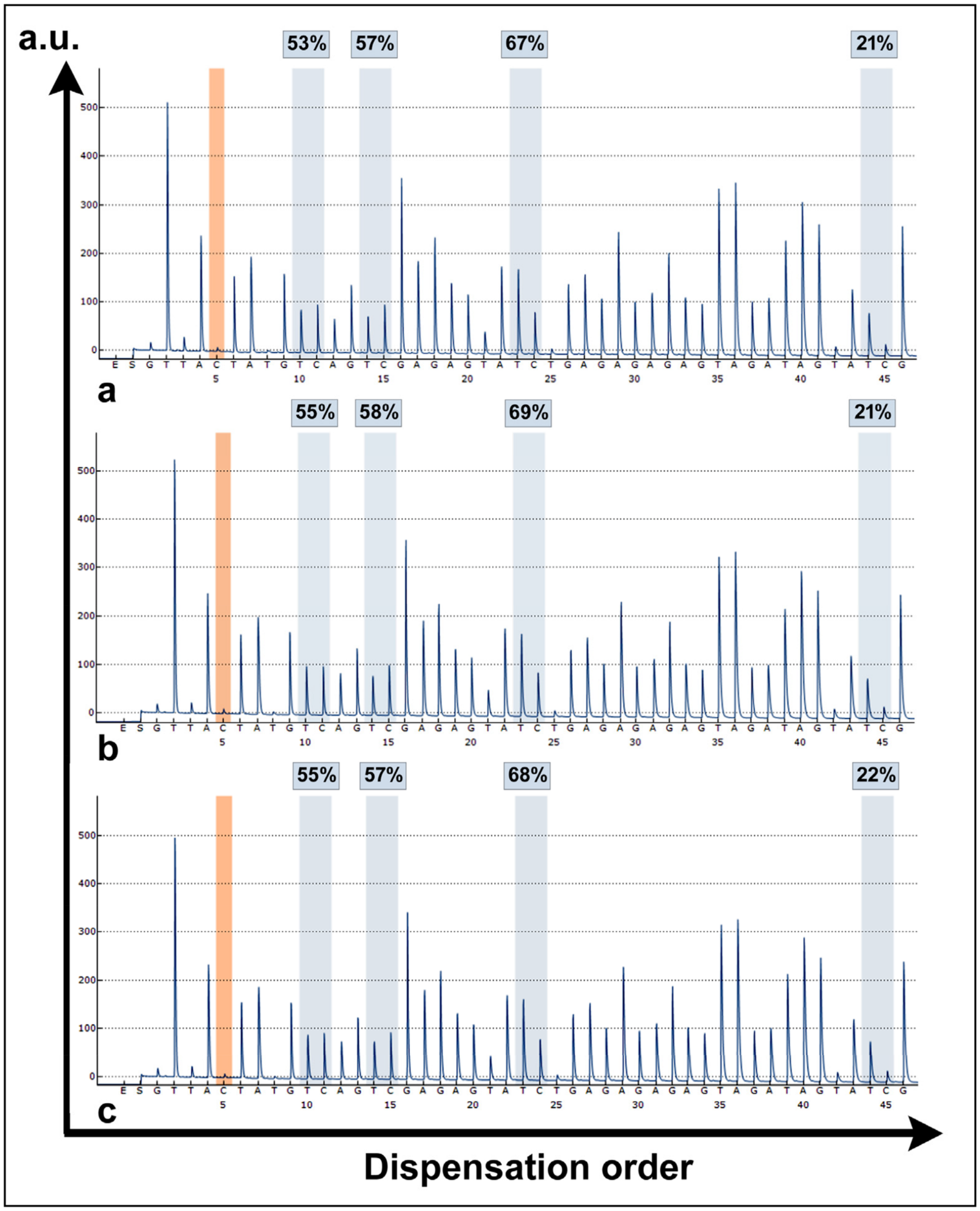

Supplementary Figure S7c: The change in methylation level at a distant (> 5 kb) subtelomeric region in Chr. Xp. Changes in methylation were quantitatively determined by pyrosequencing at four CpG sites at a randomly chosen distant subtelomeric region in Chr. X (Chr.X:37,5698-37,5741). The sequenced site is 10.2 kb distant from the adjacent TTAGGG site. Y-axis denotes signal intensity in arbitrary units (a.u.), while x-axis denote nucleotide dispensation order. The methylation level at each CpG site of the sequenced region was observed in the mock-transfected-illuminated **a.**, co-transfected; non-illuminated **b.**, and co-transfected; blue light exposed **c.** cells. Methylation level changed by 2% at CpG-1, and CpG-3 upon co-transfection, but did not increase further upon light treatment. Insignificant changes in methylation levels were also observed at the remaining CpG sites.

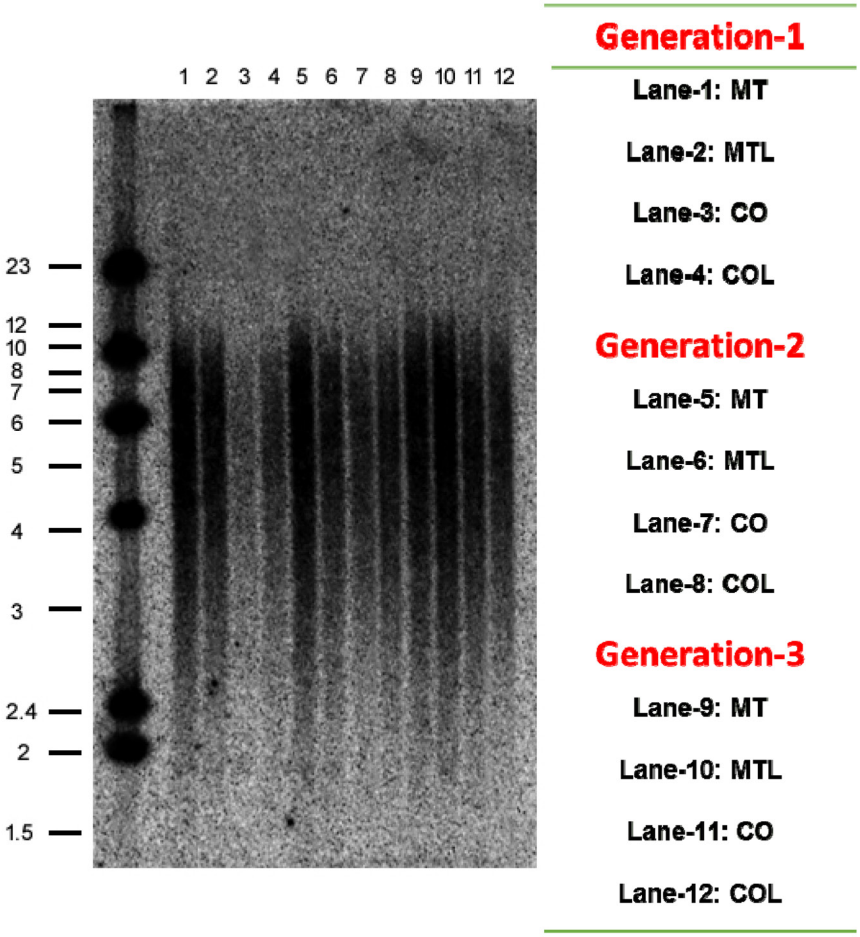

Supplementary Figure S8a: Southern Blotting to determine telomere length. Telomere length (TRF) was determined from four treatment groups namely mock-transfected (MT), mock-transfected light treated (MTL), co-transfected with TCON and ECON (CO), and co-transfected light treated (COL) by using Southern Blot based analysis. The mean telomere length (TRF) in MT and MTL groups did not show any significant change after three consecutive rounds of treatment. In contrast, changes in TRF in light treated group (COL) were significantly ($p < 0.05$) different, compared to the CO group at the end of 2nd and 3rd generations.

(*Continued*)

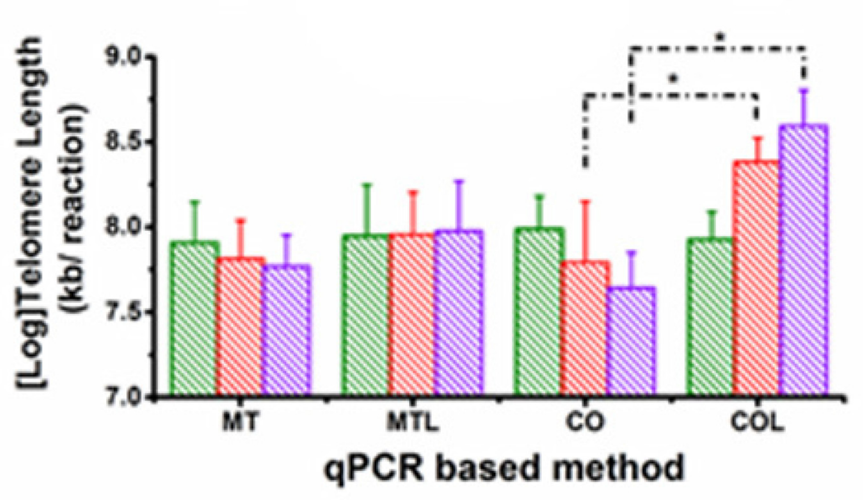

Supplementary Figure S8b: qPCR to determine telomere length. The mean telomere length (TRF) in MT and MTL groups did not show any significant change after three consecutive rounds of treatment. In contrast, changes in TRF in light treated group (COL) were significantly ($p < 0.05$) different, compared to the CO group at the end of 2nd and 3rd generations.

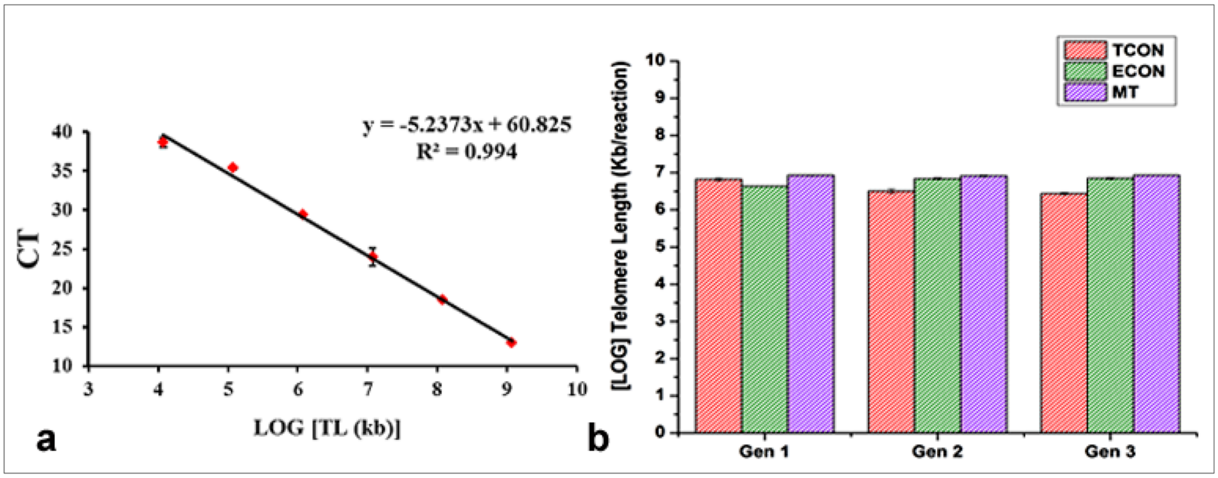

Supplementary Figure S9: Determining telomere length in TCON and ECON transfected cells. a. A standard curve for calculating length of telomere sequence (TL) per reaction tube was established followed by a standard qPCR method (O'Callaghan et al. 2008). The x-axis represents amount of telomere sequence in kb per reaction. Correlation coefficient within the linear range was 0.99. The value generated from the experimental samples using this standard curve was equal to [LOG] kb/reaction of telomere sequence per sample. **b.** Telomere length progressively decreased for three consecutive generations among the cells individually transfected with TCON. In contrast insignificant change in telomere length was observed for the ECON transfected cells at the end of Gen-2 or Gen-3. Results correspond to the mean value and S.D. of three independent measurements (n=3).

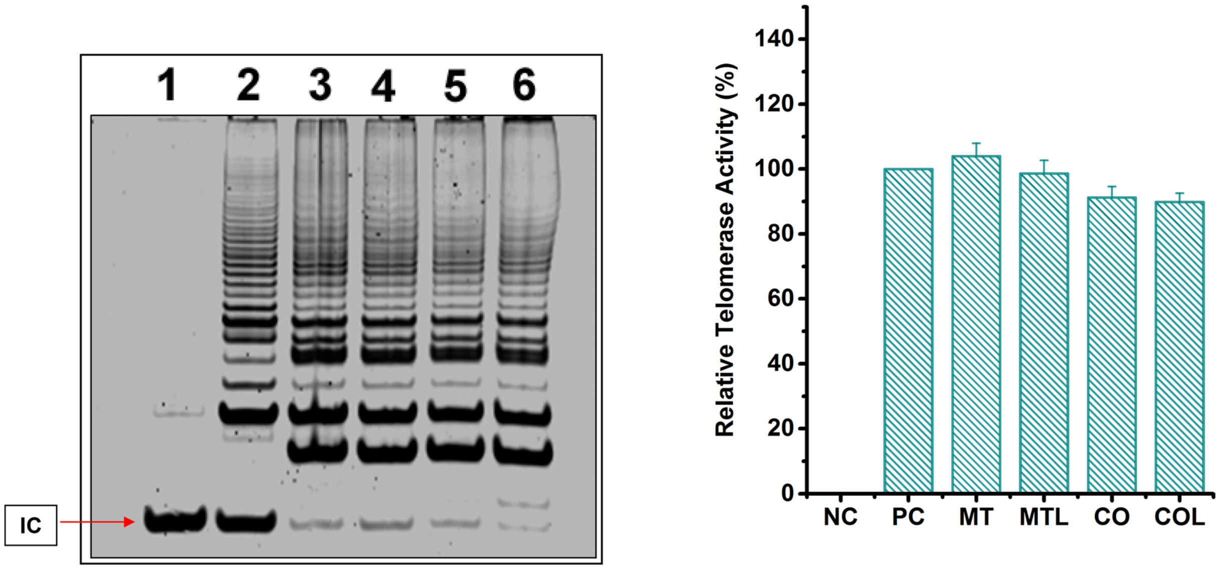

Supplementary Figure S10: Telomerase activity in both control and treated cells was determined using the telomere repeat amplification protocol (TRAP) assay. Heat inactivated lysate of the positive control was used as a negative control (NC) (lane-1) and extracts from telomerase positive cells was used as a positive control (PC) (lane-2) The control and treatment groups, including mock transfected (MT; lane-3), mock transfected-light exposed (MTL; lane-4), co-transfected (CO; lane-5) and co-transfected-light exposed (CoL; lane-6) cell lysates produced a telomerase positive characteristic 6 bp ladder, which was expected as the internal control (IC). The percentage of relative telomerase activity (RTA) was determined according to the gel image (left panel). The RTA % deviate slightly among the samples, but no statistically significant difference (p >0.10) was observed.

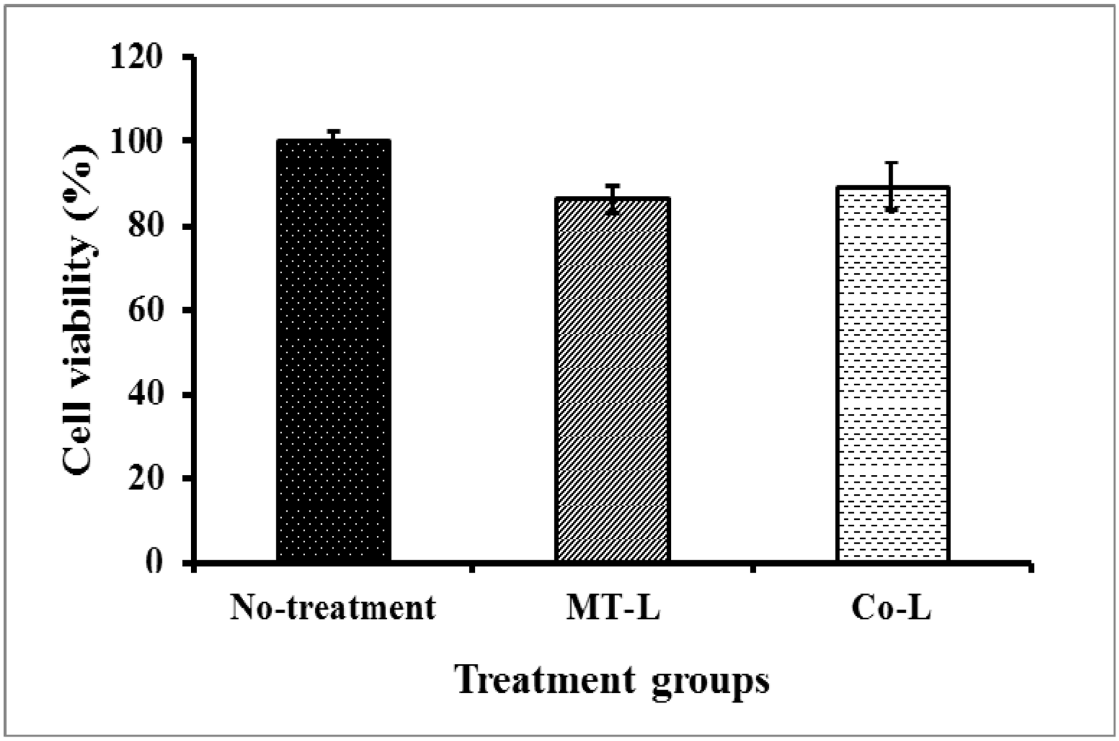

Supplementary Figure S11: Determining cytotoxicity of blue light and co-transfection of the fusion protein constructs (TCON and ECON) in HeLa cells using MTT assay. Each group of cells was treated for three consecutive generations, and the percentage of cell viability was assessed at the end of 3rd generation of treatment. Cell viability was evaluated as 86% for the light exposed mock transfected (MT-L) and 89 % for the light exposed co-transfected (Co-L) cells. Results correspond to the mean value and S.D. of three independent measurements (n=3).

Supplementary Table S1: Primers used in this study for PCR amplification of the inserts

Insert name		Forward	Reverse
TCON	**EGFP**	aatctagtGAATTCATGGT GAGCAAGGGCGAGG	tctacaaaTCTAGAgcCTTG TACAGCTCGTCCATGC
	**TRF1**	atcgttCCTGCAGGaAGCCCC AAGAAGAAGAGAAAGG TGGAGGCCAGCGGTAGGGATGCCGACCCTAC	atgtaaGCGGCCGCtcaGTCTT CGCTGTCTGAGGAAATCAG
ECON	**DNMT3A**	tgacgcGAATTCGgGtcagcgaatacaggcgca ATGCCCGCCATGCCCTCCAGC	aatcgaTCTAGActgccactagtccCCACA CACGCAAAATACTCCTTCAGCG
	**CRY2-mCherry**	cgatgcTCTAGAgagcggaagtacacccgca ATGAAGATGGACAAAAAGACTATAGTTTGGT	acttaaGCGGCCGCCTACTT GTACAGCTCGTCCATGC

The restriction sites are highlighted in yellow; linker molecules are presented in red and the nuclear localization signal (NLS) is presented in green font. All primers listed are 5' to 3'.

Supplementary Table S2: Primers used in this study for sequencing the fusion-protein constructs

TCON (EGFP-CIB1-TRF1) fusion construct		ECON (DNMT3A-CRY2-mCherry) fusion construct	
**Primer name**	**Sequence (5' to 3')**	**Primer name**	**Sequence (5' to 3')**
**Hctf**	CGGGTCTGAATCTGCTGCAAGAAG	**Hctf**	CGGGTCTGAATCTGCTGCAAGAAG
		**DCM_SP1**	TACTACATCAGCAAGCGCAAG
**ECT_SP1**	ACTACCTGAGCACCCAGTCC	**DCM_SP2**	AACAAGCCCATGATTGAATGG
**ECT_SP2**	ACGAAGGAATTGGAGAAAACG	**DCM_SP3**	TACATTGCCTCGGAGGTG
**ECT_SP3**	TCAATTGAAAAGGAACATGACAA	**DCM_SP4**	ATCTTATGGTGCACTGAAATGG
**ECT_SP4**	ACTCTCATGTGCAGAATCTCTATG	**DCM_SP5**	AAGGGCAAACCTTTTACGAGT
**Primer name**	**Sequence (3' to 5')**	**DCM_SP6**	ATCCTTGGCTGGCAGTATATC
**T7 Terminator**	GGTTATGCTAGTTATTGCTCAGCGGT	**Primer name**	**Sequence (3' to 5')**
		**T7 Terminator**	GGTTATGCTAGTTATTGCTCAGCGGT

Supplementary Table S3: Primers used in this study for bisulfite converted PCR (BSP). All primers listed are 5' to 3'

	Forward	Reverse	Product size (bp)	Annealing Temp.™ (°C)
**Sub-telomeric CpGs proximal to TTAGGG (< 5 kb)**				
**7q**	AGTGGATATTTAGGTTTA TGATTGTAAT	**5'-/5BiosG/**CTCCCCAAAAAACA CTTTACTCTTTCACA	257	58
**8q**	TGTTTTTTTTTTTTTA TTAAAAGGTGAGTT	**5'-/5BiosG/**AAAAATCTCCAAA AAACCCTTATAAAC	139	56
**16p**	GTTTTAATTGGTTTTTGA TTTTGATTAT	**5'-/5BiosG/**CTCAACACAAAC CTAAAAATCACC	164	56.5
**18p**	**5'-/5BiosG/**GTTGTGGTGT AGGTTTATAATTT	ACCCCCAAAAAAAAACT ACAAAAACC	185	56
**21q**	TTTTGGAGGATTGTGTT TAGATAGATAT	**5'-/5BiosG/**CCCAACTCCTTCCA AATAAAT	137	58
**Xp**	GTGGGTTAGTTTGTAT TGAGGTAGGTAT	**5'-/5BiosG/**AAAACCCCCT AACCCCTCC	307	57*
**Sub-telomeric CpGs distal to TTAGGG (> 5 kb)**				
**21q**	AAGATTTTGGAGTTTGA GTTAGAAG	**5'-/5BiosG/**ACTTATCCAAAA ATACCACCTACTAT	109	59
**Xp**	AGGGATTTATTGAAA ATGGAAGTAT	**5'-/5BiosG/**TTTATACCATTT AAACTTAACTTTTTTCTA	158	57
**Centromere adjacent region**				
**HSPA13 (Chr.21)**	AGGATTTTAGGGAAT TTATTTAAGAT	CTCAAAACAAACCAACTA CATTTCCC	154	56

5'-/5BiosG/ is the 5'-biotinylation on the forward or reverse BSP primers. *Xp subtelomere region was amplified in presence of Q-solution (QIAGEN).

Supplementary Table S4: Analyzed sequence and the sequencing primers for pyro-sequencing; all primers listed are 5' to 3'

Proximal Sub-telomeric region	Sequence to analyze	Primer sequence
**7q**	AAATTTGTTATYGTAGTGGTYGGATTTTTTTGGGTT ATTYGAGTGGAGYGGTYGGGTT	TTTATGATTGTAATTTTA TAATA
**8q**	YGGGTAYGTAGTTAGYGTTYGYGATT ATTYGTTTATAAGGGTTTTTT	ATTTTTATTAGG AAAGGTGT
**16p**	AYGGTTGTAATYGGGAAAGATTTTATTTAT TGTTAATGYGTTTYGAGTTGTTTTAAAGTT AGGTAGTGTTTTTAAYGTTTGTGTTTAGTAGAATGTTGTTT	ATTGGTTTTTGATTT TGATTATTT
**18p**	AACCRCCCTCRCAACCCAAATAACCRATTTAT CCTACCCRAAAAACATCR CCCTCCTCCRC	CTAAACCCCCCCTT CTACCCCTA
**21q**	TTYGTAGGTGTGYGYGGYGTTYGTA AGTGGTTAGTATAAYGTYGGGYGAA	GTTTAGATAGATATTG GTTTTTTTA
**Xp**	TYGAGGTGATTYGGGTGG GAGGTGTATYGTYGTTTATT	GAGGTAGGTATGGGT
**Distal Sub-telomeric region**	**Sequence to analyze**	**Primer sequence**
**21q**	YGYGGGTGTGTTAAGTAG GYGGAGGTYGTTATTG	GTTAGAAGAATAGTTGT TTTAGTAT
**Xp**	TTTTATAGYGYGGGAGGAGATYGAGAAG AGGAGTTTAAAGATTAAAGGGAYGGGTT	GGATTTATTGAAAATG GAAGTATT
**Centromeric region**	**Sequence to analyze**	**Primer sequence**
**HSPA13**	TAGTTTYGTYGAATAGGT TTGTGATGATTGTATTAGAYGT GAGGTATYGTTTTTATTTTTTTYG GTAGTTTYGTAGTTTTTGGGAAATGTAGTT	AAGATAGTTATTTTT TTGGTTATTA

Y/R (complement of Y) are the potential methylated cytosines at the target CpG sites.
